# Characterization of a bloom-associated alphaproteobacterial lineage, ‘*Candidatus* Phycosocius’: insights into freshwater algal-bacterial interactions

**DOI:** 10.1038/s43705-023-00228-6

**Published:** 2023-03-11

**Authors:** Yuuhiko Tanabe, Haruyo Yamaguchi, Masaki Yoshida, Atsushi Kai, Yusuke Okazaki

**Affiliations:** 1 Biodiversity Division, National Institute for Environmental Studies, Ibaraki, 305-8506 Japan; 2 Algae Biomass and Energy System R&D Center, University of Tsukuba, Ibaraki, 305-8572 Japan; 3 Institute for Chemical Research, Kyoto University, Kyoto, 611-0011 Japan

**Keywords:** Bacterial genomics, Water microbiology

## Abstract

Marine bacterial lineages associated with algal blooms, such as the *Roseobacter* clade, have been well characterized in ecological and genomic contexts, yet such lineages have rarely been explored in freshwater blooms. This study performed phenotypic and genomic analyses of an alphaproteobacterial lineage ‘*Candidatus* Phycosocius’ (denoted the *Ca*P clade), one of the few lineages ubiquitously associated with freshwater algal blooms, and described a novel species: ‘*Ca*. Phycosocius spiralis.’ Phylogenomic analyses indicated that the *Ca*P clade is a deeply branching lineage in the *Caulobacterales*. Pangenome analyses revealed characteristic features of the *Ca*P clade: aerobic anoxygenic photosynthesis and essential vitamin B auxotrophy. Genome size varies widely among members of the *Ca*P clade (2.5–3.7 Mb), likely a result of independent genome reductions at each lineage. This includes a loss of tight adherence pilus genes (*tad*) in ‘*Ca*. P. spiralis’ that may reflect its adoption of a unique spiral cell shape and corkscrew-like burrowing activity at the algal surface. Notably, quorum sensing (QS) proteins showed incongruent phylogenies, suggesting that horizontal transfers of QS genes and QS-involved interactions with specific algal partners might drive *Ca*P clade diversification. This study elucidates the ecophysiology and evolution of proteobacteria associated with freshwater algal blooms.

## Introduction

In marine environments, algal blooms often occur in early spring to early summer in the stratified euphotic zone, where nutrients are replenished annually through winter mixing. As such, marine algal blooms constitute part of marine biogeochemical cycles [1]. Each algal cell or colony in a marine bloom develops a phycosphere. This phycosphere attracts many heterotrophic bacteria that utilize dissolved organic matter, such as polysaccharides, that are rarely available in the open oligotrophic ocean [2]. Among the marine phycosphere bacteria, the *Roseobacter* clade is one of the most abundant bacterial groups in the pelagic environment and plays a significant role in global carbon and sulfur cycling [1]. Members of the *Roseobacter* clade are associated with a broad taxonomic range of algae, including dinoflagellates, diatoms, and haptophytes [1, 3]. Aside from its affinity to phytoplankton, a prominent characteristic of the *Roseobacter* clade is its metabolic versatility. These adaptations include anoxygenic photosynthesis, sulfur and carbon monoxide oxidations as sources of energy (chemolithotrophy), and algae-derived dimethylsulfoniopropionate (DMSP) assimilation [3]. In addition, members of the *Roseobacter* clade show chemotaxis toward algae or algae-derived dissolved organic matter and become sessile on the nutrient-rich algal cell surface. This is often called a ‘swim-or-stick’ lifestyle [4] and is facilitated by flagellar assembly and type IV pili expression [5]. These behaviors are controlled by a suite of phosphorelays centered on a global transcriptional regulator, CtrA, with quorum-sensing (QS) also involved in its regulation [6].

In freshwater systems, algal blooms often occur in response to increasing nitrogen and phosphorus inputs resulting from human activity, although the occurrence of blooms predates human history [7]. Climate change is also thought to accelerate freshwater blooms [7]. Freshwater bloom-forming algae include *Cyanobacteria* (e.g., *Microcystis*, *Dolichospermum*, and *Aphanizomenon*), diatoms [8], and on rare occasions, green algae (e.g., *Botryococcus braunii*) [9]. Cyanobacterial blooms are known to produce cyanotoxins that are health hazards for both humans and wildlife [7]. Many freshwater bloom-forming algae form carbohydrate-rich colonies that contribute to a variety of phycospheres. A growing number of studies have revealed that *Alphaproteobacteria*, *Gammaproteobacteria*, and *Bacteroidetes* are major constituents of freshwater phycosphere bacterial communities [10–14]. However, ecophysiological interactions between freshwater bloom-forming algae and their phycosphere bacteria have been poorly described. One study suggested the presence of metabolic complementation between *Microcystis aeruginosa* and *Roseomonas* spp. [13]. Another study suggested that *B. braunii* supplemented biotin to *Brevundimonas* sp., which in return protected *B. braunii* from colonization by other harmful bacteria [14].

A previous study isolated a novel alphaproteobacterium, ‘*Candidatus* Phycosocius bacilliformis,’ from a bloom of *B. braunii*, a colony-forming green alga (*Chlorophyta*) [15]. *B. braunii* is unique in that it accumulates hydrocarbons within the colony and has thus attracted attention as a feedstock for biofuel production [15]. ‘*Ca*. P. bacilliformis’ 16S rDNA clustered with two bacterial sequences recovered from *Microcystis* cultures, and similar sequences (showing >99.4% DNA similarity) were frequently detected in *Botryococcus* and *Microcystis* blooms collected from lakes in Asia and Africa [15]. A subsequent metagenomic study of *Microcystis*-associated bacteria in 14 US lakes revealed that relatives of ‘*Ca*. P. bacilliformis’ were detected in all 46 *Microcystis* colonies and accounted for up to 19% of bacterial abundance in the *Microcystis* phycosphere [16]. These results suggest that ‘*Ca*. P. bacilliformis’ and its relatives represent a ubiquitous lineage closely associated with freshwater phytoplankton populations. Interestingly, ‘*Ca*. P. bacilliformis’ possesses genes for bacteriochlorophyll *a* (BChl *a*), suggesting that this species is a photosynthetic bacterium [17]. However, knowledge of this lineage is limited due to the poor availability of genome sequences.

Previous research observed another bacterium, morphologically distinct from ‘*Ca*. P. bacilliformis,’ co-occurring with ‘*Ca*. P. bacilliformis’ in a nonaxenic culture of *B. braunii* [15]. ‘*Ca*. P. bacilliformis’ was rod-shaped, whereas the novel bacterium was spiral in shape (Fig. S1). In this study, the spiral bacterium was cultivated, characterized, and designated BOTRYCO-1. Analysis of 16S sequences revealed that BOTRYCO-1 was closely related to ‘*Ca*. P. bacilliformis,’ motivating further characterization of ‘*Ca*. Phycosocius.’ The BOTRYCO-1 genome sequence was obtained and used in a pangenome analysis of four ‘*Ca*. Phycosocius’ relatives. Together, these bacteria represented a unique alphaproteobacterial lineage characterized by photoheterotrophy and close association with freshwater phytoplankton blooms.

## Materials and methods

### Cultures

*B. braunii* Ba10, a nonaxenic isolate recovered from a *B. braunii* bloom in a reservoir in Myanmar [15], was used to cultivate a spiral bacterium, designated BOTRYCO-1. A two-membered culture of a bacterium and *B. braunii* Ba10 was established by inoculating algal-free bacterial suspension (obtained by filtration of Ba10 using a Minisart® syringe filter, pore size 1.2 μm, Sartorius, Göttingen, Germany) into a bacteria-free culture of Ba10 (ref. 15). The culture was maintained in liquid AF-6 medium [18] at 25 °C under continuous white light (50 μmol photon m^−2^s^−1^). The detailed cultivation protocol was as described previously [15].

### Light and electron microscopic studies

Light microscopy was performed using an IX71 microscope with a DP72 camera (Olympus, Tokyo, Japan). Catalyzed reporter deposition-fluorescence *in situ* hybridization (CARD-FISH) was performed using a BAG645 probe specific to the *Ca*P clade [15], as previously described [15]. Scanning electron microscopy (SEM) was performed using a JSM-6330F field emission SEM (JEOL, Ltd., Tokyo, Japan). Transmission electron microscopy (TEM) was performed using a TEM H-7650 (Hitachi, Tokyo, Japan). Sample preparation was as previously described for SEM [19] and TEM [15].

### Whole genome analysis

BOTRYCO-1 cells were collected by Minisart® filtration of 80 mL of a two-membered liquid culture comprising BOTRYCO-1 and *B. braunii* Ba10. Genomic DNA was extracted using NucleoBond AXG columns with buffer set III (Macherey-Nagel, Düren, Germany). Whole-genome sequencing of BOTRYCO-1 was performed using the MiSeq platform (Illumina, San Diego, CA, USA) as previously described [17]. De novo assembly of short reads was performed using SPAdes ver. 3.14.1 (ref. 20), and assembled scaffolds were polished using Pilon ver 1.24 (ref. 21). After removing experimental contaminants (based on lower K-mer coverage and BLAST analysis), the remaining scaffolds were annotated using dFAST [22]. Genome completeness was assessed using CheckM [23]. Average nucleotide (ANI) and amino acid identities (AAI) were calculated using online ANI and AAI calculators [24].

### Phylogenetic analyses

Genome sequences related to BOTRYCO-1 were retrieved from GenBank, and a phylogenomic tree was constructed using PhyloPhlAn 3.0 (ref. 25) using amino acid sequences of 400 universal marker genes (PhyloPhlAn database) [26]. The statistical confidence of each tree branch was evaluated using the bootstrap method with 100 replicates. The PufL and PufM sequences of type strains of the *Caulobacterales* related to BOTRYCO-1 were retrieved from GenBank and aligned using Clustal W [27]. Maximum likelihood (ML) phylogenetic analyses were performed using RaxML-NG ver. 1.0.1 (ref. 28). Modeltest-NG ver. 0.1.6 (ref. 29) was used to select the amino acid substitution model for ML phylogenetic reconstruction.

### Pangenome analyses and functional assessment of coding sequences

Pangenome analyses were performed using the GET_HOMOLOGUES pipeline [30], and the result was visualized using ‘ggplot2’ in R ver. 4.0.5. Initial functional annotation of proteins was performed using eggNOG mapper v.2 (ref. 31). Using the outputs of the eggNOG mapper, KEGG pathway reconstruction [32] was used to infer metabolic pathways present for each genome. Enzyme presence was further inspected by individual BLAST searches.

## Results and discussion

### Phenotypic and phylogenetic characterization of a novel species of ‘Ca. Phycosocius’

**Fig. 1:**
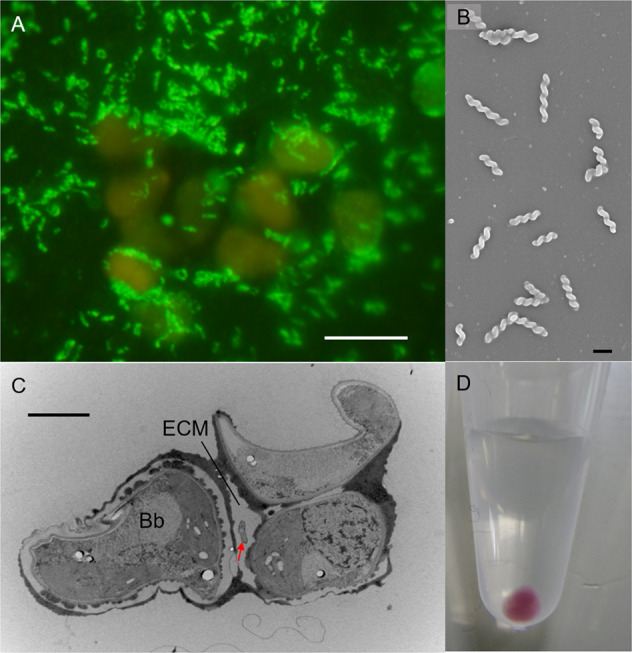
Micrographs of BOTRYCO-1. **A** CARD-FISH image of the two-membered culture of *B. braunii* (orange) and BOTRYCO-1 (yellow-green). Scale bar, 10 μm. **B** SEM photo of BOTRYCO-1 showing a spiral shape. Scale bar, 1 μm. **C** TEM photo showing a BOTRYCO-1 cell inside the extracellular matrix (ECM) of *B. braunii*. Arrow indicates BOTRYCO-1. Bb, *B. braunii*. Scale bar, 2 μm. **D** Collected cells of BOTRYCO-1 in a 1.5 mL microtube.

TEM microscopy of *B. braunii* Ba10 indicated the presence of spiral bacteria inside the extracellular matrix (ECM; Fig. S2). This differed from ‘*Ca*. P. bacilliformis,’ which is not seen inside the ECM [15]. The spiral bacterium, BOTRYCO-1, showed no growth in a low nutrient medium in which ‘*Ca*. P. bacilliformis’ had exhibited transient growth (Table S1). A culture exclusively containing *B. braunii* and BOTRYCO-1 was established, and BOTRYCO-1 growth was abundant in cocultivation (Fig. 1A), reaching > 10^4^ cells/μL during the early stationary growth phase of *B. braunii*. BOTRYCO-1 was observed actively swimming in a spiral manner in the liquid medium (Movie S1) and was aggressively motile toward *B. braunii*. BOTRYCO-1 burrowed through the ECM in a back-and-forth spiral motion near the surface of the *B. braunii* colony (Movie S2). Numerous nonmotile spiral cells were present deep inside the ECM, suggesting that, after successful colonization, the bacterium stopped moving and presumably lost its motility, reminiscent of the ‘swim-or-stick’ lifestyle of the *Roseobacter* clade [4]. SEM microscopy indicated that BOTRYCO-1 was 1.2–2.6 μm long (Fig. 1B), and TEM examination of the coculture confirmed that BOTRCYO-1 was present inside the ECM (Fig. 1C). Collected BOTRYCO-1 cells were burgundy-red (Fig. 1D), similar to those of ‘*Ca*. P. bacilliformis’ [15].

**Fig. 2:**
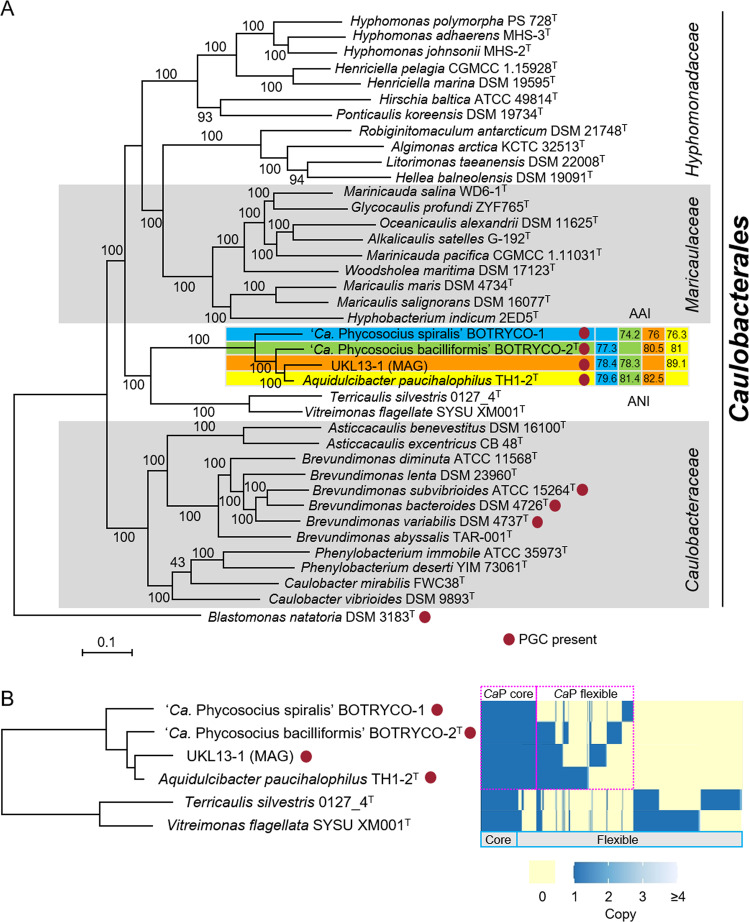
Pangenome analysis of ‘*Ca*. Phycosocius’ and relatives. **A** ML phylogenomic tree rooted with *Blastomonas natatoria*. ML bootstrap values (>80%) are indicated at each branch. Pairwise ANI and AAI values were indicated at the cluster, including ‘*Ca*. P. spiralis.’ **B** Distribution of orthologous gene clusters in the *Ca*P clade. A heatmap indicates the presence and absence of orthologs. Core and flexible genomes indicate orthologs present in all six genomes and less than six genomes of *Ca*P bacteria, respectively. *Ca*P core and flexible indicate orthologs in all and less than four *Ca*P genomes, respectively.

BLAST analysis indicated that the BOTRYCO-1 16S rDNA sequence had 98.9–99.3% sequence similarity to about a dozen bacterial sequences, including those of ‘*Ca*. P. bacilliformis,’ the recently described species *Aquidulcibacter paucihalophilus* TH1-2^T^ [33], and a metagenomic assembled genome UKL13-1 recovered from an *Aphanizomenon flos-aquae* cyanobacterial bloom [34]. Given the morphological dissimilarities (Table S1), the close relationships observed between BOTRYCO-1 and these two species were unexpected. Phylogenetic analysis of 16S rDNA indicated that these sequences, including the BOTRYCO-1 sequence, formed a monophyletic clade, hereafter denoted the *Ca*P (*Candidatus* Phycosocius) clade (Fig. S3A, B). Notably, almost all sequences of this clade were recovered from isolates or colonies of freshwater microalgae (Table S1, Fig. S3B). Our literature survey identified *Ca*P bacteria in freshwater phycosphere microbiomes worldwide (Table S2). Furthermore, analyses of global *Microcystis* microbiome data [12] revealed that *Ca*P bacteria were distributed worldwide and were one of the dominant groups of bacteria (>1% relative abundance) in 4 of 12 *Microcystis* blooms examined (Table S3). These results confirm the hypothesis that the *Ca*P clade is ubiquitously associated with freshwater blooms of broad taxonomic groups of algae [15, 16]. The *Ca*P clade and two monospecific genera, *Vitreimonas* [35] and *Terricaulis* [36], constituted a deeply branching lineage of the *Caulobacterales* both in phylogenomic and 16S rDNA trees, although the branching order within the *Ca*P *clade* was slightly different (Fig. 2A, Fig. S3A). ‘*Ca*. P. bacilliformis’ 16S rDNA had 100% similarity to *A. paucihalophilus*; however, ANI and AAI values between the four genomes in the *Ca*P clade were <95% (Fig. 2A), which is a proposed cutoff value used for bacterial species delineation [37, 38]. This suggests that each member of the *Ca*P clade represents a different species. ANI values between *A. paucihalophilus* and ‘*Ca*. P. bacilliformis’ (81.4%), and *A. paucihalophilus* and BOTRYCO-1 (79.6%), were higher than the mean genus cutoff value (73.9 %) [39], indicating that ‘*Ca*. P. bacilliformis’ and BOTRYCO-1 may be affiliated with the genus *Aquidulcibacter*. However, the colony colors of BOTRYCO-1 and ‘*Ca*. P. bacilliformis’ were purplish, different from the yellowish color of *A. paucihalophilus* (Table S1) and from those of other known *Caulobacteraceae* species [36]. In addition, species affiliated with the *Caulobacterales* have various cell shapes, including rod, vibrioid, and fusiform, but, other than BOTRYCO-1, spiral-shaped cells have not been described [40]. On the basis of its distinct cell morphology and its genetic and phenotypic similarity to ‘*Ca*. P. bacilliformis,’ we tentatively propose ‘*Candidatus* Phycosocius spiralis’ for BOTRYCO-1. Hereafter, ‘*Ca*. P. spiralis’ is used for BOTRYCO-1.

### Aerobic anoxygenic photosynthesis (AAnP) in the CaP clade

Whole-genome assembly of ‘*Ca*. P. spiralis’ produced 46 contigs with an N50 value of 149 808 bp, with the longest contig being 351 315 bp and with total contig length of 2.5 Mb. CheckM analysis indicated 100% completeness and 0% contamination, suggesting that the estimated genome size approximated the true genome size of ‘*Ca*. P. spiralis,’ and that the recovered scaffolds covered almost all genes. The estimated genome size differed from other members of the *Ca*P clade, with ‘*Ca*. P. spiralis’ having the smallest genome and *A. paucihalophilus* having the largest (3.7 Mb; Table S1). To infer the genomic basis underlying the phenotypic characteristics of *Ca*P bacteria, pangenome analyses, including the two most closely related species of the *Ca*P clade, *Terricaulis silvestris* and *Vitreimonas flagellata*, were performed. Results indicated that each *Ca*P bacterium possessed a substantial number of proteins that were not shared with other *Ca*P bacteria (Fig. 2B). The differences in genome size and protein contents among the species in the *Ca*P clade (Fig. 2B) were explained either by genome reduction or by multiple large-scale horizontal gene transfers (HGT). Accumulating evidence favors the former because the increasing association between bacteria and other organisms has often driven genome reduction in bacterial associates [41, 42]. Regardless, the genome content heterogeneity among *Ca*P bacteria likely results from independent adaptive evolution in response to different environments such as diverse bacteria-host algae interactions.

**Fig. 3:**
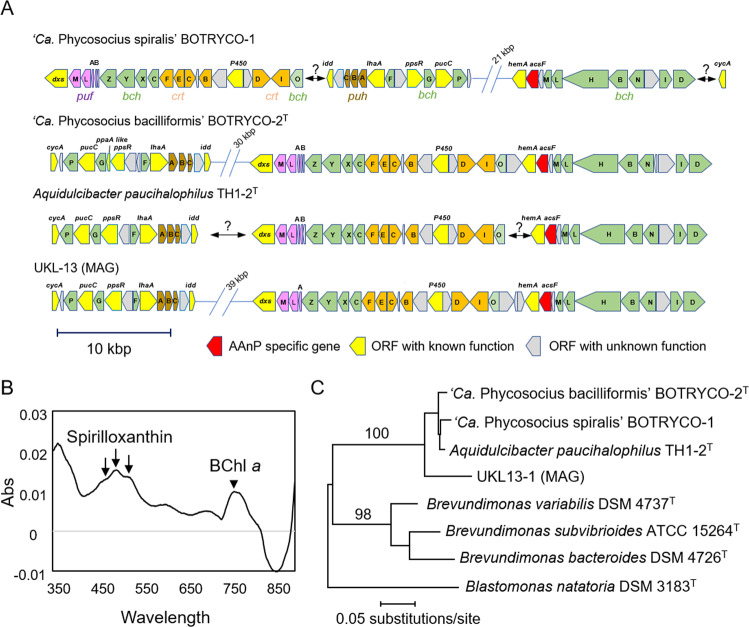
Evidence for AAnP in ‘*Ca*. Phycosocius’ and relatives. **A** Organization of PGC in the *Ca*P clade. **B** Spectral analysis of methanol extract of photosynthesis pigments in ‘*Ca*. P. spiralis.’ Arrows and an arrowhead indicated putative absorbance peaks of spirilloxanthin [44] and bacteriochlorophyll *a* (BChl a*)*, respectively. The protocol is described in Supplementary Methods. **C** ML phylogenetic tree of concatenated amino acid sequences of PufL and PufM. The tree was generated based on CPREV + G4 + F amino acid substitution model using a gap-free alignment (578 amino acids). ML bootstrap values (>80%) based on 1000 replicates are indicated at each branch.

Genome annotation of ‘*Ca*. P. spiralis’ revealed the presence of a complete set of genes for BChl *a*-based photosynthesis. These include genes for BChl a (*bch*) and carotenoid syntheses (*crt*), and light-harvesting and reaction center proteins (*puf* and *puh*). These genes were also found in the genome of ‘*Ca*. P. bacilliformis’ and other genomes of the *Ca*P clade, while being completely absent in *T. silvestris* and *V. flagellata* (Table S4). The photosynthesis genes in the *Ca*P clade were located in the photosynthetic gene cluster (PGC) and were divided between at least two subclusters separated by 20–40 kbp (Fig. 3A). Gene contents within the PGCs resembled those of other phototrophic alphaproteobacteria [43]. A characteristic feature of the PGC in the *Ca*P clade is the lack of *crtA*. This encodes spheroidene monooxygenase, which catalyzes the synthesis of spheroidenone, a photosynthetic carotenoid. By contrast, the PGCs of the *Ca*P clade contain the complete set of genes for synthesis of another carotenoid spirilloxanthin [44]. Consistent with the presence of gene sets for two photosynthetic pigments, absorption peaks indicative of BChl *a* and spirilloxanthin were detected in the spectral data of ‘*Ca*. P. spiralis’ (Fig. 3B). The purplish colony colors of ‘*Ca*. P. spiralis’ (Fig. 1D) and ‘*Ca*. P. bacilliformis’ [15] are typical for photosynthetic alphaproteobacteria [45] and are consistent with their photosynthetic potential. The yellow colony color of *A. paucihalophilus* (Table S1) suggests that yellow pigmented carotenoids are expressed in this species [46].

**Fig. 4:**
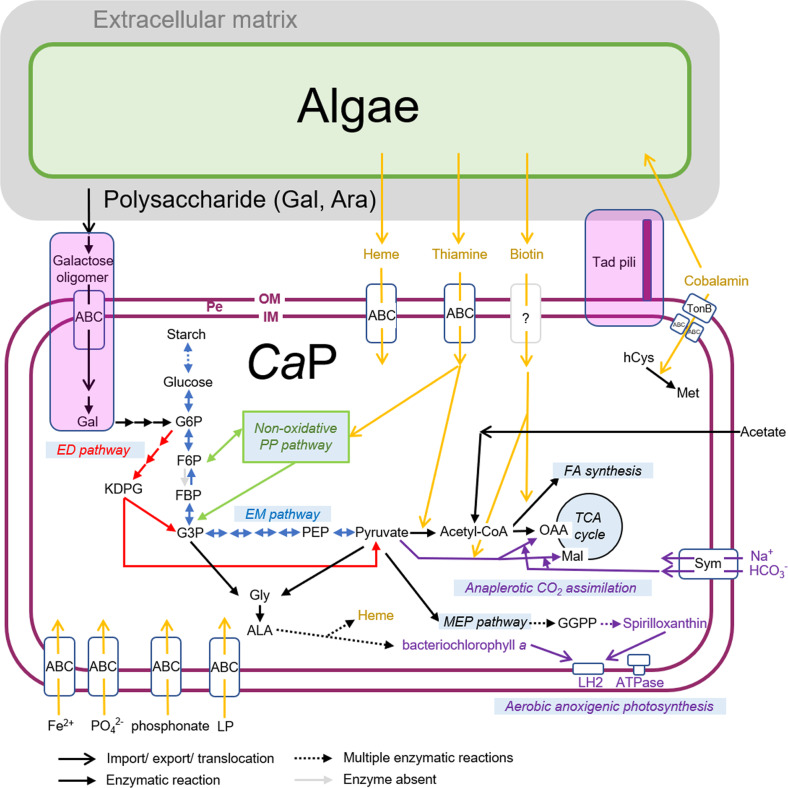
Metabolic modeling of *Ca*P clade bacteria. Features not shared in all *Ca*P bacteria are indicated in the lavender box. Note that phosphofructokinase is absent in *Ca*P bacteria (indicated by a gray arrow). PE periplasmic space, OM outer membrane, IM inner membrane, Gal galactose, Ara arabinose, G6P glucose-6-phosphate, F6P fructose 6-phosphate, FBP fructose-1,6-bisphosphate, G3P glyceraldehyde-3-phosphate, PEP phosphoenolpyruvate, OAA oxaloacetate, Mal malate, FA fatty acid, hCys homocysteine, Met methionine, Gly glycine, Ala alanine, GGPP geranylgeranyl diphosphate, Sym symporter, LP lipoprotein.

Comparison of PGCs among members of the *Ca*P clade indicated that each of the PGC genes were highly similar, and gene organization was highly conserved (Fig. 3A), suggesting common ancestry for the PGCs of the *Ca*P clade. One exceptional difference was the lack of *pufB* in the UKL-13 genome (Fig. 3A). The absence of either *pufA* or *pufB* in the PGC of alphaproteobacteria was reported previously [47]. *pufA* and *pufB* encode light-harvesting protein B-875 α and β chains, respectively [48]. Given the structural similarities between *pufA* and *pufB*, the *pufA* product likely compensates for the function of the *pufB* product in ULKL-13. Notably, all the *Ca*P genomes contained an aerobic oxidative cyclase gene, *acsF*, within the PGC as well as an oxygen-dependent coproporphyrinogen-III oxidase gene, *hemF*, both of which are hallmarks of AAnP [49, 50]. Their anaerobic counterparts, *bchE* and *hemN*, were not found in *Ca*P genomes (Table S4). Calvin-Benson cycle enzymes (e.g., RuBisCO) were also absent in *Ca*P genomes, whereas proteins involved in anaplerotic CO_2_ assimilation, phosphoenolpyruvate carboxylase, and malate dehydrogenase were present (Fig. 4 and Table S4). The latter two enzymes catalyze the formation of oxaloacetate and malate by incorporating the carboxyl group from bicarbonate into phosphoenolpyruvate and pyruvate, respectively [51]. A gene encoding a sodium-dependent bicarbonate transporter (Sbt) was found in the genomes of the *Ca*P clade (Fig. 4 and Table S4), and this was likely involved in bicarbonate uptake for anaplerotic CO_2_ assimilation. Genes for lithoautotrophy were not found in the genomes of the *Ca*P clade (Table S4). Together, these results suggest that *Ca*P members are aerobic anoxygenic photoheterotrophic bacteria.

Comprehensive genome mining suggested that the PGC is rare in the *Caulobacterales*; only three type strains of *Brevundimonas* species harbor a PGC (Fig. 2A). This sporadicity can be explained either by HGT and/or by repeated loss of the gene cluster across the *Caulobacterales*. The phylogenomic tree (Fig. 2A) and *pufLM* genealogy of the *Caulobacterales* (Fig. 3C) were concordant. Recent phylogenetic analysis of *Brevundimonas* showed that *Brevundimonas bacteroides* was most closely related to *B. variabilis*, whereas *B. subvibrioides* was more closely related to other *Brevundimonas* species that lacked a PGC [52]. This favors the HGT of PGC between the *Ca*P clade and *Brevundimonas*, although repeated losses of PGC in the *Caulobacterales* cannot be ruled out. The HGT hypothesis is further supported by frequent HGT of PGC in AAnP bacteria [53].

### Metabolic features of ‘Ca. Phycosocius’ and relatives

#### Central carbon metabolism

All members of the *Ca*P clade share the same nonoxidative pentose phosphate and glycolysis (Entner-Doudoroff) pathways, but the Embden-Meyerhof pathway is incomplete due to the absence of phosphofructokinase (Fig. 4). This type of glycolysis is often found in Gram-negative bacteria, in which the Embden-Meyerhof pathway is adopted for gluconeogenesis [54, 55] and is appropriate for *Ca*P bacteria because photosynthetic ATP production can reduce the need for glycolysis [56].

Differences in culturability in synthetic media (Table S1) suggest that *Ca*P bacteria have different nutritional requirements. Consistent with this, genes involved in galactose oligomer metabolism and transport (*ganPQ, cycB*, and *msmX*) [57] are present in ‘*Ca*. P. bacilliformis’ and *A. paucihalophilus* but are absent in ‘*Ca*. P. spiralis’ (Fig. 4, Table S4 and Fig. S4). The presence of these genes in ‘*Ca*. P. bacilliformis’ corresponds with galactose as the major component of the ECM in *B. braunii* [58]. ‘*Ca*. P. bacilliformis’ and *A. paucihalophilus* likely utilize galactose oligomers as one of their carbon sources, whereas ‘*Ca*. P. spiralis’ utilizes different carbon sources for growth.

#### Metabolism related to algal-bacterial interactions

Thiamine and biotin are essential B vitamins (VBs) for virtually all living organisms and are needed by enzymes involved in aldehyde and carboxyl group translocation, respectively. VB intercomplementation is one of the metabolic highlights of algal-bacterial mutualism [59]. *Ca*P bacteria lack genes essential for thiamine and biotin synthesis. Growth experiments indicated that axenic *B. braunii* could grow without thiamine and biotin [14], suggesting that *B. braunii* can synthesize both these VBs de novo. Putative transporters involved in thiamine uptake were detected in the genomes of *Ca*P bacteria (Table S4), but biotin transporters are yet to be identified. *Ca*P bacteria obtain VBs from associated algae (Fig. 4), other coexisting microbes, or both. Consistent with the former hypothesis, biotin uptake from *B. braunii* by phycosphere bacteria has been observed previously [14], although the transporters involved in VB export in *B. braunii* remain to be identified.

Cobalamin functions as a cofactor for methyl-group transfer in several metabolic reactions. A hallmark of the cobalamin requirement is its role as a cofactor in methionine synthesis [60]. *Ca*P bacteria encode a cobalamin-dependent methionine synthase (MetH) but do not encode cobalamin-independent methionine synthase (MetE). *Ca*P bacteria also possess a set of genes for cobalamin transport, including TonB-dependent cobalamin transporter (BtuB), a periplasmic cobalamin-binding protein (BtuF), and two putative ABC transporters for cobalamin (BtuC and BtuD; Fig. 4 and Table S4). However, cobalamin synthesis genes were absent in *Ca*P bacteria. These observations suggest that *Ca*P bacteria utilize cobalamin produced by bacteria coexisting in the *B. braunii* phycosphere.

Members of the *Roseobacter* clade are known to synthesize bioactive secondary metabolites, such as tropodithietic acid and roseobacticide, to protect or kill associated algae [61]. Genes for tropodithietic acid and roseobacticide productions (*tda*) were not found in *Ca*P bacteria (Table S4). In addition, polyketide synthase and nonribosomal peptide synthetases, which are responsible for the production of many bioactive compounds in bacteria, were not found in *Ca*P bacteria. Nonribosomal peptide synthetases were found in several genomes of the *Roseobacter* clade [62, 63]. One of their products, indigoidine, was shown to act against several competing algal pathogens, including *Vibrio* spp. [64]. Interactions between marine algae and *Roseobacter* bacteria via these bioactive compounds may govern the rise and fall of marine algal blooms [61]. In this context, the lack of bioactive compounds, either beneficial or harmful to algae, in *Ca*P bacteria is unexpected. This is indicative of a different type of interaction between *Ca*P bacteria and competing microbes and associated algae compared to bacteria of the *Roseobacter* clade. Unlike the *Roseobacter* clade, genes for DMSP assimilation were not found in *Ca*P bacteria (e.g., *DmdA*, *DmdD*, and DMSP lyases) [65] (Table S4). Consistent with this, substantial DMSP production was observed only rarely in freshwater algae [66]. DMSP metabolisms involving marine algal blooms and the *Roseobacter* clade were proposed to have an essential role in marine carbon and sulfur cycles [1]. As sulfur concentrations in freshwater are much lower than in the ocean [67], freshwater algae and associated *Ca*P bacteria are less likely to have developed similar interactions via sulfur compounds.

#### QS and cell motility

Genes for QS were identified in all *Ca*P bacterial genomes, as would be expected from the frequent observation of QS in bacterial pathogens and symbionts of eukaryotes [68]. Autoinducer synthase (LuxI) and response regulator (LuxR), a pair of proteins involved in QS [69], are present in ‘*Ca*. P. spiralis’ and ‘*Ca*. P. bacilliformis’, both of which have a green algal partner (Table S4). *A. paucihalophilus* and UKL-13, recovered from cyanobacteria, lack LuxI and contain only one LuxR. Such orphan LuxRs likely respond to exogenous QS molecules produced by coexisting microbes [68]. QS systems not only are involved in bacterial colonization of the algal surface [5] but also induce algal responses such as growth promotion or inhibition [70]. It is therefore possible that different modes of QS signaling might help to specify the algal partners of *Ca*P bacteria, and this can be elucidated by examination of host specificities for the different *Ca*P bacteria. Phylogenetic analyses revealed discordant and unresolved phylogenies of LuxI and LuxR (Fig. S5A, B), suggesting complex evolutionary histories of QS genes, including within the *Ca*P clade. The phylogenetic complexity can most easily be explained by HGT of QS genes. This is consistent with previous research suggesting that HGT events involving QS genes are frequent in proteobacteria [68]. Therefore, HGT of QS genes might have altered *Ca*P bacterial preferences for specific algae, which in turn may have led to the divergence of the *Ca*P clade.

Host-bacterial colonization is governed by chemotaxis [71], and it is therefore reasonable to assume that the ‘aggressive’ motility of ‘*Ca*. P. spiralis’ toward *B. braunii* (Movie S2) occurs in response to molecules produced by the alga. This is supported by the presence of genes for chemotaxis in all *Ca*P bacteria, namely, *cheA*, *cheB*, *cheD*, *cheR*, *cheW, cheX*, *cheY*, and *cheZ* in a *che* operon, and 2–4 chemotaxis receptor proteins (methyl-accepting chemotaxis proteins, MCP; Table S4). Different MCPs bind to different compounds, thereby directing chemotaxis [72]. Molecules implicated in bacterial chemotaxis toward algae include DMSP, amino acids, sugars, and carbohydrates [71]. At present, it is difficult to predict a chemical ligand for the *Ca*P MCPs due to their low sequence similarities to characterized MCPs.

Genes involved in flagellum expression were found in all *Ca*P bacteria (Table S4), although flagella were not observed in ‘*Ca*. P. spiralis.’ It is possible that flagella were expressed in hidden life stages, or that technical limitations hampered their observation. In the *Caulobacterales*, flagellar assembly is synchronized with the cell cycle, which is controlled by >20 regulatory proteins, including CtrA, PleC/D, and DivJ/K [73]. All *Ca*P bacteria possess most of these regulatory genes (Table S4), suggesting that a similar regulatory pathway governs flagellar formation and the cell cycle.

### Implications for algal-CaP bacterial interactions and their ecological impacts

Previous research showed that ‘*Ca*. P. bacilliformis’ promoted the growth of the host alga *B. braunii* during its growth, indicative of a mutualistic relationship [15]. However, preliminary growth experiments indicated that ‘*Ca*. P. spiralis’ had unstable effects (sometimes positive, sometimes neutral) on the growth of *B. braunii* (Fig. S6). It is possible that bacterial abundance relative to *B. braunii* abundance in the culture determines the fate of the algal-bacterial interaction [74]. The phycosphere of *B. braunii* in natural environments harbors many microbes in addition to ‘*Ca*. P. spiralis’ and likely has multiple alga-microbe interactions. For example, a nonaxenic culture of *B. braunii* Ba10 harbored at least four species in addition to ‘*Ca*. Phycosocius’ (Fig. S7). The impact of ‘*Ca*. P. spiralis’ on the growth of *B. braunii* might therefore vary in a microbiome-dependent manner. One suggestion is that the *Roseobacter* clade and flavobacteria synergistically remineralize phytoplankton-derived organic matter in marine environments [1], and similar interactions might occur between *Ca*P and other bacteria.

Given the smaller genome size of ‘*Ca*. P. spiralis’ compared to other *Ca*P bacteria, our initial hypothesis was that ‘*Ca*. P. bacilliformis’ was ancestral to ‘*Ca*. P. spiralis.’ However, pangenome analyses suggest independent evolution for each lineage. Increasing the nutritional and metabolic dependence of ‘*Ca* P. spiralis’ on associated algae may have accelerated its genome reduction, transforming ‘*Ca* P. spiralis’ from a mutualist into a commensal. Both theoretical and empirical data suggest that symbiotic relationships are not stable and oscillate along the continuum between mutualism and parasitism [41]. In this context, *Ca*P bacteria fluctuate between mutualism and commensalism.

**Fig. 5:**
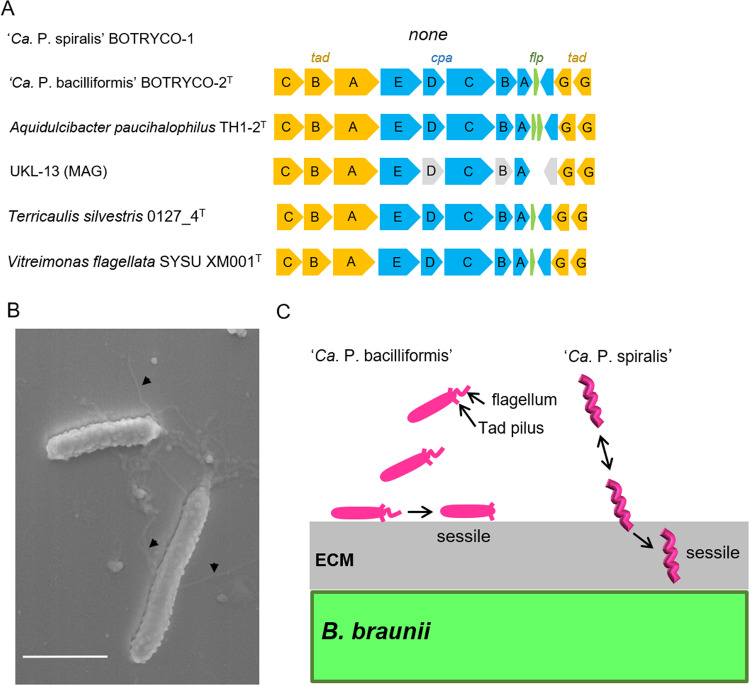
Tad pili and their possible role in algal surface attachment. **A** Tad gene cluster in the *Ca*P clade. Gray boxes indicate putative pseudogenes. **B** SEM image shows putative Tad pili in ‘Ca. P. bacilliformis’. Scale bar, 1 μm. **C** Schematic of different modes of attachment of two ‘*Ca*. Phycosocius’ species onto/into the algal surface.

One characteristic gene set that is absent in ‘*Ca*. P. spiralis’ but present in other members of the *Ca*P clade is the Tad gene cluster, which encodes tight adherence (Tad) pili (a class of type IV pili) and associated regulatory proteins (Fig. 5A) [75]. Tad pili are widespread in bacteria, including *Caulobacter*, a distant relative of the *Ca*P clade [76], and are involved in surface attachment and biofilm formation [75]. Swimming and sessile cells of ‘*Ca*. P. spiralis’ were observed off and on host algae, respectively (Movie S2). This shift in cell motility was reminiscent of the ‘swim-or-stick’ lifestyle of algal-associated members of the *Roseobacter* clade [4], which use Tad pili to attach to algal partners [77]. SEM imaging captured a putative Tad pilus-like structure in ‘*Ca*. P. bacilliformis’ in coculture with *B. braunii* (Fig. 5B). This suggests that all *Ca*P bacteria except ‘*Ca*. P. spiralis’ use Tad pili, allowing for a *Roseobacter*-like ‘swim-or-stick’ lifestyle. The loss of a Tad gene cluster in ‘*Ca*. P. spiralis’ likely coincides with its acquisition of spiral motility and ECM burrowing, which renders attachment to the host surface no longer necessary (Fig. 5C). ‘*Ca*. P. spiralis’ therefore adopts a unique strategy for its ‘swim-or-stick’ lifestyle that does not employ Tad pili for algal surface attachment. The Tad gene cluster in UKL-13 may be incomplete due to the pseudogenization of three genes, including *cpeE* and *cpeD* (Fig. 5A), and Tad pili might thus be absent in UKL-13. If so, UKL-13 might have a spiral-shaped morphology similar to that of ‘*Ca* P. spiralis.’

Several features of the *Ca*P clade, such as association with algal colonies and the metabolic potential of AAnP, point to the clade having partial ecophysiological similarity to the marine *Roseobacter* clade. Of these features, the shared presence of AAnP is of particular interest. A close correlation between marine AAnP bacteria and occurrence of phytoplankton blooms has been reported. The reason for this correlation is not yet fully understood [1, 46]; however, it has been suggested that freshwater bloom-associated AAnP bacteria play a role in maintaining redox balance in blooms via thiosulfate reduction and dissimilatory sulfate reduction pathways [12, 78]. *Ca*P bacteria are unlikely to have a similar role because the genes involved in these processes (*sox*) are absent in *Ca*P bacteria (Table S4). Field surveys indicate that AAnP bacterial abundance is positively correlated to temporal phytoplankton bloom dynamics [79], and *Ca*P members likely comprise some of these AAnP bacteria. AAnP bacteria show higher growth rates than nonphotosynthetic heterotrophs when optimal light is available due to their capacity to reduce respiration in order to produce ATP [80]. AAnP bacteria are thus preferentially selected in organic matter-rich and sunlit environments like algal blooms in both marine and freshwater environments. This suggests that sunlight exposure owing to the association with blooms is one of the driving forces for the evolution of AAnP in the *Ca*P clade. A recent report indicated that lineages in *Gemmatimonadata* associated with phytoplankton are photoheterotrophs [81]. Many species of the alphaproteobacterium genus *Methylobacterium*, which are often found on plant leaves, also possess genes for AAnP [47]. Photoheterotrophy might thus be one of the derived features in bacteria most closely associated with phototrophic organisms such as algae and plants. Conversely, close association with algae exposes associated bacteria to higher levels of sunlight-derived UV exposure. To cope with the DNA damage from UV exposure, bacteriorhodopsin-containing bacteria in marine environments, for example, possess more photolyase genes than bacteria armed with light-screening pigments [82]. With the exception of ‘*Ca* P. spiralis,’ which has a reduced genome, this is also the case with *Ca*P bacteria, which have three photolyase genes. Nonphotosynthetic *Ca*P relatives have one or no photolyase genes (Table S4).

In conclusion, this study highlights the ecological and physiological characteristics of members of the *Ca*P clade, which have adopted photoheterotrophic and VB auxotrophic growth in close association with algal blooms in freshwater. At least two species of the *Ca*P clade, ‘*Ca*. P. bacilliformis’ [15] and ‘*Ca*. P. spiralis,’ stick to the algal colony and subsequently become sessile. With the exception of VB auxotrophy, these ecophysiological features resemble those of the marine *Roseobacter* clade. In this context, the *Ca*P clade is a freshwater counterpart of the *Roseobacter* clade. However, the *Ca*P clade is genetically far less diverse (99% 16S rDNA sequence divergence) than the marine *Roseobacter* clade, which includes >50 genera encompassing ~89% 16S rDNA sequence diversity [1, 83]. This suggests that the *Ca*P clade may have derived much later than the *Roseobacter* clade, although the possibility cannot be excluded that the diversity of the *Ca*P clade was underestimated in this study owing to the poor availability of *Ca*P genomes. Future studies include (1) unraveling the genomic and metabolic diversity of the *Ca*P clade by including more strains; (2) exploring molecular interactions between *Ca*P bacteria and their algal partners, including QS signaling; (3) investigating detailed population dynamics of *Ca*P bacteria in relation to algal bloom dynamics; and (4) elucidating the metabolic regulation and balance between heterotrophic and phototrophic energy acquisitions of *Ca*P bacteria. These studies will shed light on the ecological impact of the *Ca*P clade on carbon cycling during algal blooms in freshwater environments. These studies will also allow examination of the hypothesis that evolution of photoheterotrophy in bacteria was driven by close association with phototrophic organisms, including microalgae.

## Supplementary Information

Supplementary Movie S1

Supplementary Movie S2

Supplementary information

## Data Availability

Genome sequence data of ‘*Ca*. P. spiralis’ BOTRYCO-1 are available at GenBank under the accession number BPFZ01000000.
