# Supplementary material for: Characterization of a bloom-associated alphaproteobacterial lineage, ‘Candidatus Phycosocius’: insights into freshwater algal-bacterial interactions
Source: ISME Commun. 2023 Mar 11;3:20. doi: 10.1038/s43705-023-00228-6 (PMC10008586; doi:10.1038/s43705-023-00228-6)
Supplement: Supplementary file 3 — Supplementary information [file 43705_2023_228_MOESM3_ESM.pdf]

Supplementary information

Characterization of a bloom-associated alphaproteobacterial lineage '*Candidatus* Phycosocius': Insights into freshwater algal-bacterial interaction

Yuuhiko Tanabe[1,2)*], Haruyo Yamaguchi[1)], Masaki Yoshida[2)], Atsushi Kai[2)], Yusuke Okazaki[3)]

[1)] Biodiversity Division, National Institute for Environmental Studies, 305-8506, Ibaraki, Japan

[2)] Algae Biomass and Energy System R&D Center, University of Tsukuba, 305-8572, Ibaraki, Japan

[3)] Institute for Chemical Research, Kyoto University, 611-0011, Kyoto, Japan

*For correspondence: ytanabehiko@gmail.com

Table S1. Phenotypic characteristics

Strain	Genome size (Mb)	Genome completeness[a]	Cell shape	Source	Cell (colony) color	Growth on		
						TSA[b]	R2A	NA[c]
'*Ca.* Phycosocius spiralis' BOTRYCO-1	**2.5**	**100%**	spiral	*Botryococcus braunii* (*Chlorophyta*)	burgundy-red	-	NT	-
'*Ca.* Phycosocius bacilliformis' BOTRYCO-2[T] [15]	**3.3**	**100%**	rod	*Botryococcus braunii* (*Chlorophyta*)	burgundy-red	-	NT	+/-[d]
Aquidulcibacter paucihalophilus TH1-2[T] [33]	**3.7**	**100%**	rod	species undescribed (*Cyanobacteria*)	yellow	+	+	NT
UKL-13 (MAG) [34]	**3.5**	**complete**	N/A	*Aphanizomenon flos-aquae* (*Cyanobacteria*)	N/A	N/A	N/A	N/A
Terricaulis silvestris 0127_4[T] [36]	**3.8**	**complete**	rod	forest soil	white	NT	NT	NT
Vitreimonas flagellata SYSU XM001[T] [35]	**3.8**	**100%**	coccobacilli	activated sludge	light pink	-	+	-

[a] Based on CheckM analysis. 'Complete' means that the complete genome sequence is available.

[b] Trypticase soy agar.

[c] Nutrient agar.

[d] Transient growth in 1/10 diluted NA [15].

Table S2. Literature survey for *Ca*P bacteria from phycosphere metagenomic data

Data	Source	Dominant phytoplankton	*Ca* P abundance	Taxonomy or ID assigned in the study	Reference
MAG	*Microcystis* colony, China	*Microcystis*	ND	DF05	Li *et al.* (2019)
16S amplicon	Cyanobloom, Canada	*Dolichospermum, Microcystis*	ND	Phycosocius baciliformis	Tromas *et al.* (2017)
16S amplicon	Desmid-rich reservoir water, Czechia	*Staurastum planktonicum*	ND	UKL-13	[80]
16S amplicon	Cyanobloom, Norway	*Dolichospermum, Aphanizomenon*	0.65% [a]	OTU30, OTU152	Parulekar *et al.* (2017)
MAG	*Microcystis* colony, Canada	*Microcystis*	ND	Aquidulcibacter	[13]

ND, not documented.

MAG, metagenome-assembled genome.

[a] Supplementary data sets accompanying the publication were used for analyses of the relative abundance of *Ca*P bacteria compared to that of total bacteria, including cyanobacterial sequences.

Table S3. *Ca*P abundance in *Microcystis* bloom microbiomes

Locality	*Ca* P abundance
Aasee, Germany	0.97%
Wentowsee, Gerrmany	0.42%
Lake Villerest, France	0%
Lake Kinneret, Israel	0%
Belső-tó lake, Hungary	0.06%
Lake Chaohu, China	0.59%
Lake Taihu, China	1.51%
Lake Clarendon, Australia	0.18%
Lake Rotoehu, New Zealand	0.44%
Grand Lake, USA	1.59%
Castlerock Lake, USA	7.48%
FP23, USA	2.93%

Relative abundance of *Ca*P clade bacteria in 16S rRNA gene amplicon sequences from global *Microcystis* bloom microbiomes [12]. The calculation was based on the ASV (amplicon sequence variant) table of non-cyanobacterial sequences provided by the authors. The analysis targeted the V3-V4 regions of the 16S rRNA gene, and ASVs with up to two mismatches to the sequence of '*Ca*. P. spiralis' BOTRYCO-1 (corresponding to those taxonomically assigned to "genus UKL-13" in the study pipeline) were designated as members of the *Ca*P clade. Values from multiple biological replicates from the same sample were averaged.

Table S4 List of genes and proteins mentioned in the text.

Pathway/metabolism	Gene/protein	EC number	"*Ca*. P. spiralis" BOTRYCO-1[T]	"*Ca*. P. bacilliformis" BOTRYCO-2[T]	*A. paucihalophilus* TH1-2[T]	UKL-13 (MAG)	*Vitreimonas flagellata* SYSU XM001[T]	*Terricaulis silvestris* 0127_4[T]	CDSEARCH_top hit	blastp e-value	blastp query
Anaplerotic CO2 assimilation	PEP carboxylase	4.1.1.31	PsB1_0822	PbB2_00585	WP_085340905.1	AMS30413.1	WP_135211771.1	WP_158765183.1	PRK00009	0	PsB1_0822
	malic enzyme	1.1.1.40	PsB1_0639	PbB2_00530	WP_085338210.1	AMS30367.1	WP_135211976.1	WP_158767772.1	PRK07232	0	PsB1_0639
Photosynthesis	pufL		PsB1_0734	PbB2_00547	WP_085338188.1	AMS30384.1	-	-	PRK14506	3.21E-174	PsB1_0734
	pufM		PsB1_0735	PbB2_00546	WP_085338189.1	AMS30383.1	-	-	PRK14504	5.00E-178	PsB1_0735
	pufA		PsB1_0733	PbB2_00548	WP_085338187.1	AMS30385.1	-	-	NA	NA	PsB1_0733
	pufB		PsB1_0732	PbB2_00549	WP_085338186.1	-	-	-	pfam00556	9.20E-08	PsB1_0732
	bchB		PsB1_0832	PbB2_00573	WP_085340895.1	AMS30405.1	-	-	PRK02910	0	PsB1_0832
	bchC		PsB1_0728	PbB2_00554	WP_085338221.1	AMS30389.1	-	-	TIGR01202	2.01E-143	PsB1_0728
	bchD		PsB1_0828	PbB2_00578	WP_085340899.1	AMS30408.1	-	-	PRK13406	2.40E-159	PsB1_0828
	bchF		PsB1_0868	PbB2_00514	WP_085340010.1	AMS31115.1	-	-	pfam07284	4.06E-74	PsB1_0868
	bchG		PsB1_0865	PbB2_00509	WP_085340007.1	AMS30349.1	-	-	PRK07566	1.18E-136	PsB1_0865
	bchH		PsB1_0833	PbB2_00572	WP_232780927.1	AMS30404.1	-	-	PRK13405	0	PsB1_0833
	bchI		PsB1_0829	PbB2_00577	WP_085340898.1	AMS30407.1	-	-	PRK13407	2.53E-142	PsB1_0829
	bchL		PsB1_0834	PbB2_00571	WP_085341015.1	AMS31124.1	-	-	PRK13185	5.41E-170	PsB1_0834
	bchM		PsB1_0835	PbB2_00570	WP_085340894.1	AMS30403.1	-	-	PRK07580	7.99E-102	PsB1_0835
	bchN		PsB1_0831	PbB2_00574	WP_085340896.1	AMS30406.1	-	-	PRK02842	0	PsB1_0831
	bchO		PsB1_0717	PbB2_00565	WP_085338175.1	AMS30397.1	-	-	TIGR03056	1.37E-68	PsB1_0717
	bchP		PsB1_0863	PbB2_00507	WP_085340005.1	AMS31114.1	-	-	TIGR02023	2.00E-156	PsB1_0863
	bchX		PsB1_0729	PbB2_00553	WP_085338183.1	AMS30388.1	-	-	cd02033	0	PsB1_0729
	bchY		PsB1_0730	PbB2_00552	WP_085338184.1	AMS30387.1	-	-	TIGR02015	0	PsB1_0730
	bchZ		PsB1_0731	PbB2_00551	WP_085338185.1	AMS30386.1	-	-	TIGR02014	0	PsB1_0731
	puhA		PsB1_0870	PbB2_00516	WP_085340012.1	AMS30353.1	-	-	cd00226	2.18E-93	PsB1_0870
	puhB		PsB1_0871	PbB2_00517	WP_085340013.1	AMS30354.1	-	-	pfam03703	8.01E-05	PsB1_0871
	puhC		PsB1_0872	PbB2_00518	WP_085340014.1	AMS30355.1	-	-	TIGR03054	3.44E-28	PsB1_0872
	crtB		PsB1_0723	PbB2_00559	WP_198533994.1	AMS31121.1	-	-	cd00683	2.30E-64	PsB1_0723
	crtC		PsB1_0725	PbB2_00557	WP_198533995.1	AMS31120.1	-	-	cd21471	6.58E-120	PsB1_0725
	crtD		PsB1_0719	PbB2_00563	WP_085338177.1	AMS31122.1	-	-	TIGR02734	7.95E-60	PsB1_0719
	crtE		PsB1_0726	PbB2_00556	WP_085338181.1	AMS30391.1	-	-	cd00685	4.70E-43	PsB1_0726
	crtF		PsB1_0727	PbB2_00555	WP_085338182.1	AMS30390.1	-	-	pfam00891	9.87E-33	PsB1_0727
	crtI		PsB1_0718	PbB2_00564	WP_085338176.1	AMS30396.1	-	-	TIGR02734	0	PsB1_0718
Heme synthesis	5-aminolevulinate synthase (ALAS)	2.3.1.37	PsB1_0838	PbB2_00883	WP_085339669.1	AMS29315.1	WP_135210454.1	WP_158766167.1	PRK13393	0	PsB1_0838
	glutamyl-tRNA reductase (hemA)	1.2.1.70	-	-	-	-	-	-			WP_011337894.1
Sodium dependent bicarbonate symporter	sbt		PsB1_1468	PbB2_00667<br>PbB2_02405	WP_085338536.1<br>WP_157893695.1	AMS29143.1<br>AMS30035.1	WP_239590762.1	-	pfam05982	1.13E-113	PsB1_1468
Thiamine transporter	thiB		PsB1_1136	PbB2_01941	WP_085339463.1	AMS28732.1	WP_135212939.1	WP_158767578.1	COG1176	2.05E-79	PsB1_1136
	thiP		PsB1_1135	PbB2_01942	WP_085339464.1	AMS28731.1	WP_239590995.1	WP_228446081.1	COG1177	5.87E-83	PsB1_1135
	thiQ		PsB1_1134	PbB2_01943	WP_085339465.1	AMS28730.1	WP_135212940.1	WP_228446079.1	COG3842	1.76E-160	PsB1_1134
	pnuT		-	-	-	-	-	-			WP_011072658.1
Cobalamin transporter	btuB		PsB1_2248	PbB2_01863	WP_085339259.1	AMS29717.1	WP_135210256.1	WP_158765895.1	COG4206	9.73E-110	PsB1_2248
	btuF		PsB1_2249	PbB2_01862	WP_106407579.1	AMS29716.1	WP_167755798.1	WP_158764268.1	COG0614	9.73E-19	PsB1_2249
	btuC		PsB1_2250	PbB2_01861	WP_085339263.1	AMS29715.1	WP_135212904.1	WP_158764269.1	pfam01032	5.59E-73	PsB1_2250
	btuD		PsB1_2251	PbB2_01860	WP_085339265.1	AMS29714.1	WP_135212905.1	WP_158764270.1	COG1120	1.06E-85	PsB1_2251
Cobalamin-dependent enzymes	methylmalonyl-CoA mutase (mmuT)	5.4.99.2	PsB1_1623	PbB2_01113	WP_158766392.1	AMS28898.1	WP_135210717.1	WP_158766392.1	PRK09426	0	PsB1_1623
			PsB1_1624	PbB2_01114	WP_158766394.1	AMS28899.1	WP_135210718.1	WP_158766394.1	cd03677	1.71e-143	PsB1_1624
	metH	2.1.1.13	PsB1_0167	PbB2_00761	WP_085340536.1	AMS29215.1	WP_135211110.1	WP_158766885.1	PRK09490	0	PsB1_0167
Photosynthesis (aerobic)	acsF	1.14.13.81	PsB1_0837	PbB2_00568	WP_085340893.1	AMS30402.1	-	-	PRK13654	0	PsB1_0837
	hemF	1.3.3.3	PsB1_1643	PbB2_01748	WP_085338111.1	AMS29619.1	WP_135212198.1	WP_158764882.1	PRK05330	9.46E-159	PsB1_1643
Photosynthesis (anaerobic)	bchE	1.21.98.3	-	-	-	-	-	-			WP_200227915.1
	hemN	1.3.98.3	-	-	-	-	WP_135212803.1	-	PRK09249	0	WP_135212803.1
Tight adherence protein	tadA/cpaF		-	PbB2_02888	WP_085340992.1	AMS30490.1	WP_135211049.1	WP_158766831.1	COG4962	0	PbB2_02888
	tadB		-	PbB2_02887	WP_085340991.1	AMS30489.1	WP_135211040.1	WP_158766832.1	COG4965	1.19e-82	PbB2_02887
	tadC		-	PbB2_02886	WP_085340990.1	AMS31143.1	WP_239590796.1	WP_228445700.1	COG2064	1.59E-39	PbB2_02886
	cpaC-like		-	PbB2_02895	WP_085340999.1	-	WP_167755621.1	WP_158766824.1	pfam13629	6.96E-17	PbB2_02895
	tadG		-	PbB2_02896	WP_085341000.1	AMS31145.1	WP_135211041.1	WP_158766823.1	COG4961	2.92E-12	PbB2_02896
	tadG		-	PbB2_02897	WP_198534294.1	AMS30493.1	WP_135211050.1	WP_158766822.1	COG4961	2.63E-15	PbB2_02897
	cpaA		-	PbB2_02893	WP_085340996.1	AMS30492.1	WP_135211044.1	WP_158766826.1	COG4960	2.46E-18	PbB2_02893
	cpaB		-	PbB2_02892	WP_157893748.1	-	WP_135211045.1	WP_158766827.1	TIGR03177	4.97E-47	PbB2_02892
	cpaC		-	PbB2_02891	WP_157893747.1	AMS30491.1	WP_135211046.1	WP_158766828.1	COG4964	1.56E-126	PbB2_02891
	cpaD		-	PbB2_02890	WP_085340994.1	-	WP_167755622.1	WP_158766829.1	pfam09476	6.33E-37	PbB2_02890
	cpaE		-	PbB2_02889	WP_085340993.1	AMS31144.1	WP_135211048.1	WP_158766830.1	COG4963	2.22E-82	PbB2_02889
	flp		-	PbB2_02894	WP_085340997.1<br>WP_085340998.1<br>WP_085338196.1<br>WP_085340776.1	-	WP_135211043.1<br>WP_135211510.1	WP_158766825.1<br>WP_158767374.1<br>WP_158767225.1	COG3847	1.28E-15	PbB2_02894
Quorum sensing[a)]	luxI_1		-	PbB2_02798	-	-	-	-	COG3916	2.58E-33	PbB2_02798
	luxR_1		-	PbB2_02799	-	AMS28679.1	-	-	smart00421	1.27E-19	PbB2_02799
	luxI_2		PsB1_0502	-	-	-	-	-	COG3916	2.02E-28	PsB1_0502
	luxR_2		PsB1_0503	-	-	-	-	-	PRK13870	5.20E-14	PsB1_0503
	luxR_6 (orphan)		PsB1_1629	PbB2_01121	WP_198534062.1	-	-	-	smart00421	5.05E-20	PsB1_1629
	luxI_3		-	-	-	-	WP_135212786.1	-	COG3916	2.11E-40	WP_135212786.1
	luxR_3		-	-	-	-	WP_135212785.1	-	pfam03472	3.60E-26	WP_135212785.1
	luxI_4		-	-	-	-	WP_167755506.1	-	COG3916	5.92E-47	WP_167755506.1
	luxR_4		-	-	-	-	WP_135209718.1	-	pfam03472	3.24E-23	WP_135209718.1
	luxI_5		-	-	-	-	WP_135211269.1	-	COG3916	3.73E-48	WP_135211269.1
	luxR_5		-	-	-	-	WP_135211270.1	-	pfam03472	4.81E-24	WP_135211270.1
	luxR_7 (orphan)		-	-	-	-	WP_135210322.1	-	pfam03472	1.38E-17	WP_135210322.1
	luxR_8 (orphan)		-	-	-	-	-	WP_158765845.1	PRK10188	7.03E-27	WP_158765845.1
CtrA phosphorelay	ctrA		PsB1_0584	PbB2_00366	GBF56709.1	AMS30216.1	WP_135211658.1	WP_158764583.1	COG0745	9.46E-76	PsB1_0584
	divJ		PsB1_2193	PbB2_01208	WP_085338926.1	-	WP_239590817.1	WP_158766443.1	COG0642	2.06E-46	PsB1_2193
	pleC		PsB1_1430	PbB2_00963	WP_198534214.1	-	WP_135210735.1<br>WP_135213178.1	WP_158764560.1<br>WP_158766416.1	COG0642	2.10E-56	PsB1_1430
	pleD		PsB1_2135	PbB2_01025	WP_085339082.1	AMS29422.1	WP_135209888.1	WP_158765639.1	PRK09581	0	PsB1_2135
	popA		PsB1_1450	PbB2_03068	WP_157893631.1	-	WP_135210121.1	-	COG3706	1.49E-28	PsB1_1450
Type IV secretion	VirD2		PsB1_2162	-	WP_085338137.1<br>WP_085338358.1<br>WP_085339527.1	-	WP_135209707.1<br>WP_135210311.1<br>WP_135211271.1<br>WP_135212778.1<br>WP_167755832.1	WP_158765803.1	COG3843	6.34E-22	PsB1_2162
	VirB1		-	-	-	-	-	-			WP_010974915.1
	VirB2		-	PbB2_00490	WP_085339232.1<br>WP_085339831.1<br>WP_085341062.1<br>WP_085341148.1	AMS29730.1<br>AMS31110.1	WP_135209826.1<br>WP_135210318.1<br>WP_135212843.1	WP_158767599.1	COG3838	1.18E-10	PbB2_00490
	VirB3		-	PbB2_00489	WP_085339234.1WP_085339825.1<br>WP_085341061.1<br>WP_085341147.1	AMS30331.1<br>AMS29729.1	WP_239590700.1<br>WP_135210317.1<br>WP_135212844.1	WP_158765849.1<br>WP_158767598.1	COG3702	2.07E-28	PbB2_00489

	VirB5		-	-	WP_085339238.1<br>WP_085341059.1<br>WP_085341145.1	-	WP_135209828.1<br>WP_135212846.1	WP_158765851.1	PRK13874	1.91E-23	WP_085339238.1
	VirB7		-	-	-	-	-	-			AAL57015.1
	VirB9		-	PbB2_00485	WP_085339243.1<br>WP_085339820.1<br>WP_085341057.1<br>WP_085341142.1	-	WP_135210312.1<br>WP_135209831.1<br>WP_167755790.1	WP_158765854.1<br>WP_158767595.1	TIGR02781	5.77E-67	PbB2_00485
	VirB6		-	PbB2_00487	WP_085339240.1<br>WP_085339822.1<br>WP_085341072.1<br>WP_085341144.1<br>WP_157893757.1<br>WP_157893764.1	AMS29728.1<br>AMS30329.1	WP_167755561.1<br>WP_135209829.1<br>WP_135212847.1	WP_158765852.1<br>WP_158767596.1	pfam04610	1.49E-18	PbB2_00487
	VirB8		-	PbB2_00486	WP_085339242.1<br>WP_085339821.1<br>WP_085341058.1<br>WP_085341143.1	AMS29727.1	WP_135210313.1<br>WP_135209830.1<br>WP_135212848.1	WP_158765853.1	COG3736	8.27E-43	PbB2_00486
	VirB10		-	PbB2_00484	WP_085339245.1<br>WP_085339819.1WP_08534<br>1056.1<br>WP_085341141.1	-	WP_135210311.1<br>WP_135209832.1<br>WP_135212850.1	WP_158765855.1<br>WP_158767594.1	NF038091	4.00E-74	PbB2_00484
	VirB4		-	PbB2_00488	WP_085339236.1<br>WP_085339824.1<br>WP_085341060.1<br>WP_085341060.1	AMS30330.1	WP_135210316.1<br>WP_135209827.1<br>WP_135212845.1	WP_158765850.1<br>WP_158767597.1	COG3451	0	PbB2_00488
	VirB11		-	PbB2_00483	WP_085339231.1<br>WP_085339818.1<br>WP_085341063.1<br>WP_085341149.1	AMS29731.1<br>AMS30328.1	WP_135210319.1<br>WP_135209825.1<br>WP_135212842.1	WP_158765848.1<br>WP_158767593.1	TIGR02788	4.82E-126	PbB2_00483
	VirD4		-	-	-	-	WP_135209823.1<br>WP_135210321.1<br>WP_135212840.1<br>WP_167755846.1	WP_158765846.1<br>WP_158765451.1	PRK13876	2.06E-168	LOCUS_02080
Aromatic compound degradation	pcaGH	1.13.11.3	-	-	-	-	-	WP_158764928.1	TIGR02422	4.66E-138	WP_158764928.1
	boxC	4.1.2.44	-	-	-	-	-	-			WP_169641897.1
Carbon monoxide oxidation	coxL	1.2.5.3	-	-	-	-	-	-			WP_012177884.1
Sulfur oxidation	soxB		-	-	-	-	-	-			WP_011567768.1
Denitrification	nirS/K	1.7.2.1	-	-	-	-	-	-			WP_012179840.1
Nitrate assimilation	nasA	1.7.99.-	-	-	-	-	-	-			WP_011570215.1
Dimethylsulfoniopropionate degradation	dmdA		-	-	-	-	-	-			WP_011281570.1
	dmdD		-	-	-	-	-	-			WP_011049477.1
	acuI		-	-	-	-	-	-			WP_011047645.1
	dddD		-	-	-	-	-	-			A6W2K8.1
	dddL		-	-	-	-	-	-			WP_011336734.1
	dddP		-	-	-	-	-	-			WP_165821347.1
	dddQ		-	-	-	-	-	-			WP_011047333.1
	dddW		-	-	-	-	-	-			WP_011047333.1
Tropodithietic acid production	tdaA		-	-	-	-	-	-			WP_014881729.1
	tdaB		-	-	-	-	-	-			WP_014881728.1
	tdaC		-	-	-	-	-	-			WP_014881727.1
	tdaD		-	-	-	-	-	-			WP_014881726.1
	tdaE		-	-	-	-	-	-			WP_014881725.1
	tdaF			-	-	-	-	-			WP_061047554.1
Galactose metabolism	α-amylase		-	PbB2_02706	WP_085338432.1	-	-	-	cd00551	5.41E-53	PbB2_02706
	ganQ		-	PbB2_02707	WP_085338433.1	-	-	-	COG3833	2.11E-88	PbB2_02707
	ganP		-	PbB2_02708	WP_085338434.1	-	-	-	COG1175	6.86E-80	PbB2_02708
	cycB		-	PbB2_02709	WP_085338435.1	-	-	-	cd13657	1.70E-94	PbB2_02709
	msmX		-	PbB2_02710	WP_085338436.1	-	-	-	PRK11650	0	PbB2_02710
	galT	2.7.7.12	PsB1_0663	PbB2_01693	WP_085340615.1	AMS30617.1	-	-	COG0153	0	PsB1_0663
	galK	2.7.1.6	PsB1_0662	PbB2_01694	WP_085340616.1	AMS30618.1	-	-	COG0153	6.05E-88	PsB1_0662
	ganA	3.2.1.23	PsB1_0659<br>PsB1_0661	PbB2_01695	WP_085340617.1	AMS30619.1	-	-	pfam02449	4.53E-29	PsB1_0659
Chemotaxis[a)]	cheA		PsB1_1932<br>PsB1_1992	PbB2_00045<br>PbB2_03124	WP_085337985.1	AMS29965.1<br>AMS29908.1	WP_135211671.1	-	COG0643	0	PsB1_1932
	cheB		PsB1_1935	PbB2_00048	-	AMS29968.1	WP_135211668.1	-	PRK00742	1.84E-146	PsB1_1935
	cheB2		PsB1_1995	PbB2_03127	WP_085337987.1	AMS29911.1	-	-	PRK00742	6.87E-158	PsB1_1995
	cheD		PsB1_1937	PbB2_00050	-	AMS29970.1	WP_135211666.1	-	cd16352	2.69E-44	PsB1_1937
	cheR		PsB1_1934	PbB2_00047	-	AMS29967.1	WP_135211669.1	-	smart00138	1.78E-81	PsB1_1934
	cheR2		PsB1_1996	PbB2_03128	WP_085337988.1	AMS29912.1	WP_135211469.1	WP_158764962.1	COG1352	1.07E-89	PsB1_1996
	cheR3		-	-	-	-	WP_167755654.1	-	COG1352	1.31E-30	WP_167755654.1
	cheW		PsB1_1933	PbB2_00046	-	AMS29966.1	WP_135211670.1	-	cd00732	1.20E-39	PsB1_1933
	cheW2		PsB1_1993	PbB2_03125	WP_085337986.1	AMS29909.1	-	-	cd00732	2.38E-35	PsB1_1993
	cheX		PsB1_1930	PbB2_00043	-	AMS29963.1	-	-	pfam13466	4.28E-06	PsB1_1930
	cheX1		-	-	-	-	WP_135211673.1	-	pfam13467	1.15E-09	WP_135211673.1
	cheX2		-	-	-	-	-	WP_158764421.1	pfam13690	8.81E-07	WP_158764421.1
	cheY1		PsB1_1931	PbB2_00044	-	AMS29964.1	WP_135211672.1	-	cd17562	1.00E-60	PsB1_1931
	cheY2		PsB1_1936	PbB2_00049	-	AMS29969.1	WP_135211667.1	-	cd19923	2.41E-37	PsB1_1936
	cheY3		PsB1_1994	PbB2_03126	WP_085338012.1	AMS29910.1	WP_135211468.1	WP_158764963.1	cd19923	2.44E-30	PsB1_1994
	cheY4		PsB1_0754	PbB2_01535	WP_085339492.1<br>WP_198534138.1	AMS28288.1	-	-	cd19923	4.98E-30	PsB1_0754
	cheY5		PsB1_1837	PbB2_00124	WP_232780890.1	AMS30005.1	-	-	cd17546	1.82E-25	PsB1_1837
	cheY6		-	-	-	-	WP_135211664.1	-	pfam00072	2.57E-13	WP_135211664.1
	cheZ		PsB1_2182	PbB2_01447	WP_085338486.1	AMS28351.1	-	-	pfam04344	8.41E-08	PsB1_2182
	MCP		PsB1_1929	PbB2_00042<br>PbB2_01711	WP_085339864.1	AMS29962.1	WP_239590858.1<br>WP_135211674.1<br>WP_135212972.1<br>WP_135211026.1<br>WP_167755761.1<br>WP_135213064.1<br>WP_135212189.1<br>WP_135210103.1<br>WP_239590743.1	-	PRK15041	9.79E-60	PsB1_1929
	MCP2		PsB1_1941	PbB2_00054	-	-	-	-	smart00283	2.23E-21	PsB1_1941
	MCP3		PsB1_1193	PbB2_00213	WP_198534231.1	AMS30086.1	-	-	COG0840	1.35E-18	PsB1_1193
	MCP4		PsB1_1009<br>PsB1_1535	PbB2_00458<br>PbB2_01848<br>PbB2_01852	WP_085338710.1<br>WP_085340153.1	-	-	-	cd01068	7.74E-29	PsB1_1009
	MCP5		PsB1_1185	PbB2_00644	WP_085340956.1	AMS30461.1	-	-	cd08504	2.31E-89	PsB1_1185

		MCP6		-	-	-	-	WP_135210524.1	-	COG0642	2.04E-35	WP_135210524.1
Flagellar assembly												
	Basal body/hook	fliE		PsB1_1587	PbB2_02463	WP_085338093.1	AMS29603.1	WP_135210818.1	WP_158766506.1	PRK00732	1.41E-12	PsB1_1587
				PsB1_2211	-	-	-	WP_135212642.1	-	pfam02049	1.36E-16	PsB1_2211
		fliF		PsB1_2210	-	-	-	WP_135212641.1	-	pfam01514	1.72E-55	PsB1_2210
				PsB1_0379	PbB2_02310	WP_157893412.1	AMS28578.1	WP_239590856.1	WP_239590856.1	PRK06007	1.25E-153	PsB1_0379
		fliG		PsB1_0380	PbB2_02311	WP_085338161.1	AMS28577.1	WP_135211650.1	WP_158764595.1	PRK05686	1.50E-123	PsB1_0380
		fliH		PsB1_0381	PbB2_02312	WP_085338162.1	AMS28576.1	WP_135211651.1	WP_158764594.1	PRK06032	2.94E-31	PsB1_0381
		fliI		PsB1_2207	PbB2_00365	WP_085341291.1	AMS31077.1	WP_135212638.1	WP_158764584.1	PRK08927	0	PsB1_2207
				PsB1_0585				WP_135211657.1				
		fliJ		PsB1_0586	PbB2_00364	WP_085341290.1	AMS30215.1	WP_135211656.1	WP_158764585.1	TIGR02473	1.01E-03	PsB1_0586
		fliK		PsB1_0375	PbB2_02306	WP_085338156.1	AMS28581.1	WP_135211645.1	WP_158764600.1	cd17470	2.18E-13	PsB1_0375
		fliL		PsB1_1597	PbB2_01106	WP_085338100.1	AMS29609.1	WP_135210926.1	WP_158766643.1	PRK12785	1.18E-33	PsB1_1597
		fliM		PsB1_1596	PbB2_01105	WP_085338099.1	AMS29608.1	WP_135210925.1	WP_158766642.1	PRK12795	0	PsB1_1596
		fliN		PsB1_0382	PbB2_02313	WP_085338163.1	AMS30735.1	WP_135211652.1	WP_158764593.1	NA	NA	PsB1_0382
		fliP		PsB1_1591	PbB2_02459	WP_106407529.1	AMS30961.1	WP_239590779.1	WP_228446057.1	PRK05699	3.91E-91	PsB1_1591
				PsB1_2200				WP_135212630.1				
		fliQ		PsB1_1097	PbB2_01172	WP_085338958.1	AMS28852.1	WP_135210813.1	WP_158766502.1	PRK06010	1.57E-09	PsB1_1097
				PsB1_1630	-	-	-	WP_135212654.1	-	COG1987	1.19E-12	PsB1_1630
		fliR		PsB1_1096	PbB2_01173	WP_085338957.1	AMS28851.1	WP_135210812.1	WP_158766501.1	PRK05701	1.02E-52	PsB1_1096
		flhE		-	-	-	-		-			NP_416392
		flhA		PsB1_0384	PbB2_02315	WP_198533992.1	AMS30734.1	WP_135211654.1	WP_158764591.1	PRK06012	0	PsB1_0384
								WP_135212651.1				
		flhB		PsB1_1095	PbB2_01174	WP_085338956.1	AMS28850.1	WP_135210811.1	WP_158766500.1	PRK05702	4.45E-143	PsB1_1095
		flgA		PsB1_1601	PbB2_01109	WP_157893404.1	AMS29612.1	WP_135210929.1	WP_158766646.1	PRK12786	1.20E-29	PsB1_1601
		flgB		PsB1_1589	PbB2_02461	WP_085338116.1	AMS30960.1	WP_135210816.1	WP_228445677.1	PRK06004	5.20E-37	PsB1_1589
				PsB1_2213	-	-	-	WP_135212644.1	-	COG1815	3.35E-11	PsB1_2213
		flgC		PsB1_1588	PbB2_02462	WP_085338094.1	AMS29604.1	WP_135210817.1	WP_158766505.1	PRK05681	5.98E-51	PsB1_1588
				PsB1_2212	-	-	-	WP_135212643.1	-	COG1558	2.72E-38	PsB1_2212
		flgD		PsB1_0376	PbB2_02307	WP_085338157.1	AMS28580.1	WP_135211646.1	WP_158764599.1	COG1843	1.57E-17	PsB1_0376
				PsB1_2205				WP_135212635.1				
		flgE		PsB1_0377	PbB2_02308	WP_085338158.1	AMS28579.1	WP_135211647.1	WP_158764598.1	PRK05682	2.29E-91	PsB1_0377
		flgF		PsB1_1598	PbB2_01107	WP_085338101.1	AMS29610.1	WP_135210927.1	WP_158766644.1	PRK12689	1.63E-60	PsB1_1598
				PsB1_2218	-	-	-	-	-	PRK12690	1.26E-43	PsB1_2218
		flgG		PsB1_1599	PbB2_01108	WP_085338102.1	AMS29611.1	WP_135210928.1	WP_158766645.1	PRK12691	6.34E-159	PsB1_1599
				PsB1_2217	-	-	-	WP_135212648.1	-	TIGR03506	3.74E-44	PsB1_2217
		flgH		PsB1_1602	PbB2_01110	WP_085338104.1	AMS29613.1	WP_167755608.1	WP_158766647.1	PRK00249	6.31E-86	PsB1_1602
				PsB1_2215	-	-	-	WP_135212646.1	-	pfam02107	5.58E-37	PsB1_2215
		flgI		PsB1_1636	PbB2_01741	WP_085338117.1	AMS30963.1	WP_135213437.1	WP_158768097.1	pfam02119	4.34E-179	PsB1_1636
				PsB1_2214				WP_135212645.1				
		flgJ		PsB1_1637	PbB2_01742	WP_085338107.1	AMS30964.1	WP_135213297.1	WP_158766654.1	PRK12790	6.22E-11	WP_158766654.1
		flgK		PsB1_0374	PbB2_02305	WP_085338155.1	AMS28582.1	WP_135211644.1	WP_158764601.1	COG1256	5.98E-41	PsB1_0374
		flgL		PsB1_0373	PbB2_02304	WP_085338154.1	AMS28583.1	WP_135211643.1	WP_158764602.1	PRK08913	4.96E-12	WP_158764602.1
		flgO		PsB1_1590	PbB2_02460	WP_157893402.1	AMS29605.1	-	WP_158766504.1	NA	NA	PsB1_1590
		flgP		-	-	-	-	-	-			Rsph17029_1665
		flgQ		-	-	-	-	-	-			A0Z60_02665
		motX		-	-	-	-	-	-			QDX24845.1
		motY		-	-	-	-	-	-			B18_00595
	Filament	fliC		PsB1_2269	PbB2_02329	WP_085339582.1	AMS28565.1	WP_135213292.1	QGZ95830.1	PRK12687	7.06E-24	PsB1_2269
				PsB1_2270	PbB2_02330	WP_085340862.1	AMS28564.1					
				PsB1_2271	PbB2_02331	WP_085340449.1	AMS28563.1					
				PsB1_0491	PbB2_02332	WP_085338750.1	AMS28562.1					
				PsB1_0492	PbB2_02333	WP_085340437.1	AMS28561.1					
				PsB1_0493	PbB2_02334	WP_085341288.1	AMS29850.1					
				PsB1_0519	PbB2_01370	WP_085341287.1	AMS29849.1					
				PsB1_0520	PbB2_01371	WP_085339023.1	AMS29848.1					
				PsB1_2274	PbB2_00359		AMS29847.1					
					PbB2_00360		AMS28441.1					
					PbB2_02567		AMS28366.1					
					PbB2_03009		AMS30211.1					
					PbB2_03045		AMS30210.1					
							AMS30732.1					
		fliD		-	-	-	-	-	-	-		AYV44800.1
		fliS		-	-	-	-	-	-	-		AOW22391.1
		fliT		-	-	-	-	-	-	-		QLD75692.1
	Stator	motA		PsB1_0654	PbB2_01701	WP_085340623.1	AMS30624.1	WP_135212380.1	WP_158765110.1	PRK09110	3.88E-149	PsB1_0654
		motB		PsB1_0033	PbB2_00893	WP_085339679.1	AMS29322.1	WP_135210460.1	WP_158766175.1	PRK09041	7.81E-46	PsB1_0033
		motC		-	-	-	-	-	-			ACS38400.1
		motD		-	-	-	-	-	-			CAX22262.1
	Regulator	flgM		-	-	-	-	-	-			ATI81378.1
		flgN		-	-	-	-	-	-			QLD78187.1
		fliX		PsB1_1635	PbB2_01740	WP_085338106.1	AMS29614.1	-	-	PRK12787	6.19E-28	PsB1_1635
		fliY		-	-	-	-	-	-			AYV48356.1
		fliZ		-	-	-	-	-	-			NP_416431.2
		flrC		PsB1_0383	PbB2_02314	WP_085338164.1	AMS28575.1	WP_135211653.1	WP_158764592.1	COG2204	6.30E-164	PsB1_0383
		fliA		-	-	-	-	-	-			AOW25464.1
Lyase[a)]		photolyase PhrB1	4.1.99.3	-	PbB2_00037	WP_085338007.1	AMS29958.1	WP_135210116.1	-	COG0415	2.01E-27	PbB2_00037
					PbB2_00904	WP_085340069.1	AMS29332.1					
		photolyase PhrB2	4.1.99.3	PsB1_0024	PbB2_00038	WP_085338008.1	AMS29959.1	-	-	COG0415	0	PsB1_0024

[a)] Different paraloques are arbitrarily numbered.

[b)] Lux proteins with no counterpart in the adjacent region are indicated with '(orphan)'.

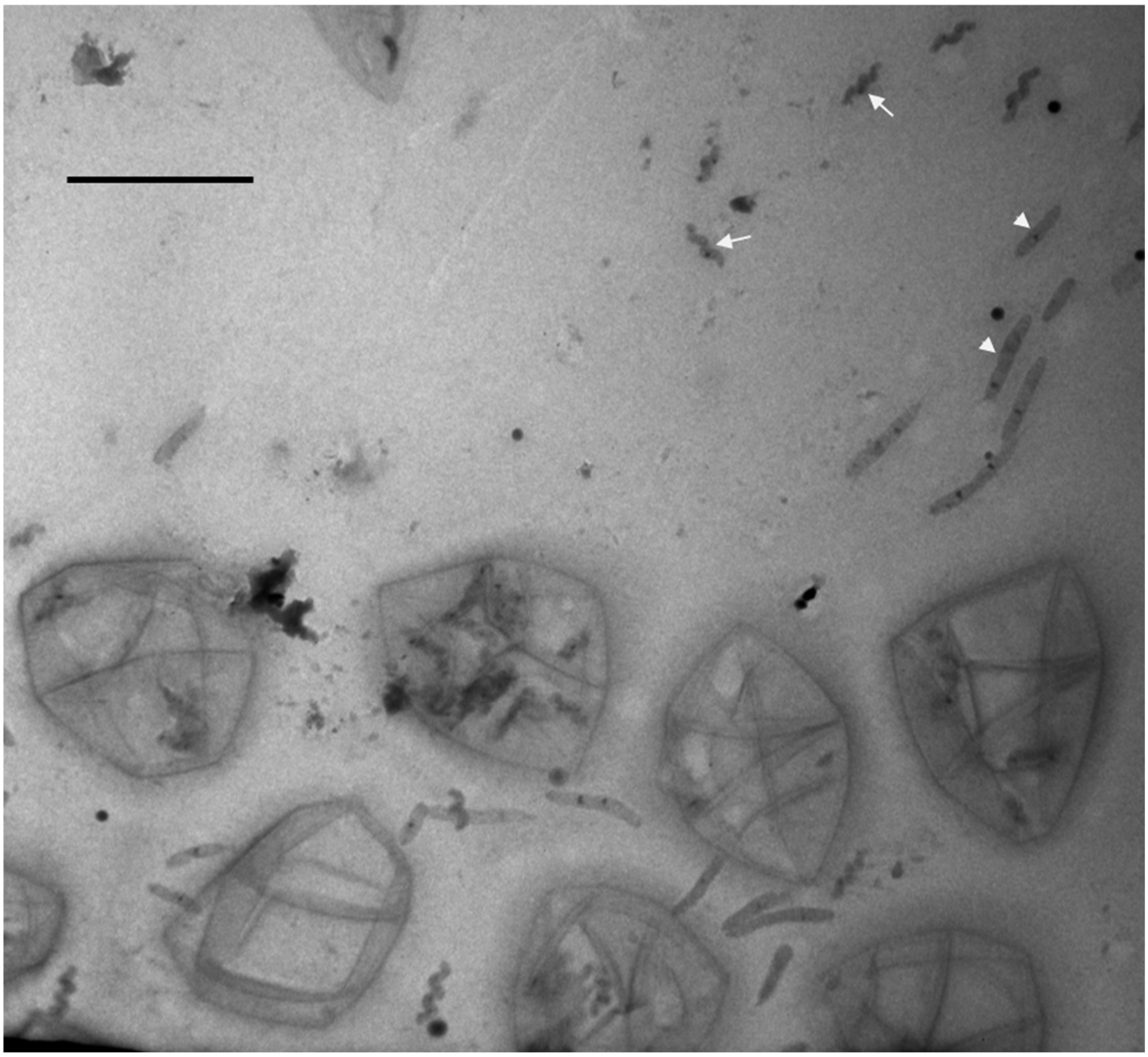

Fig. S1 Whole mount TEM image of *Botryococcus braunii* Ba10. Arrows, spiral bacteria; arrowheads, rod-shaped bacteria (‘*Ca.* Phycosocius bacilliformis’). Scale bar, 5 µm

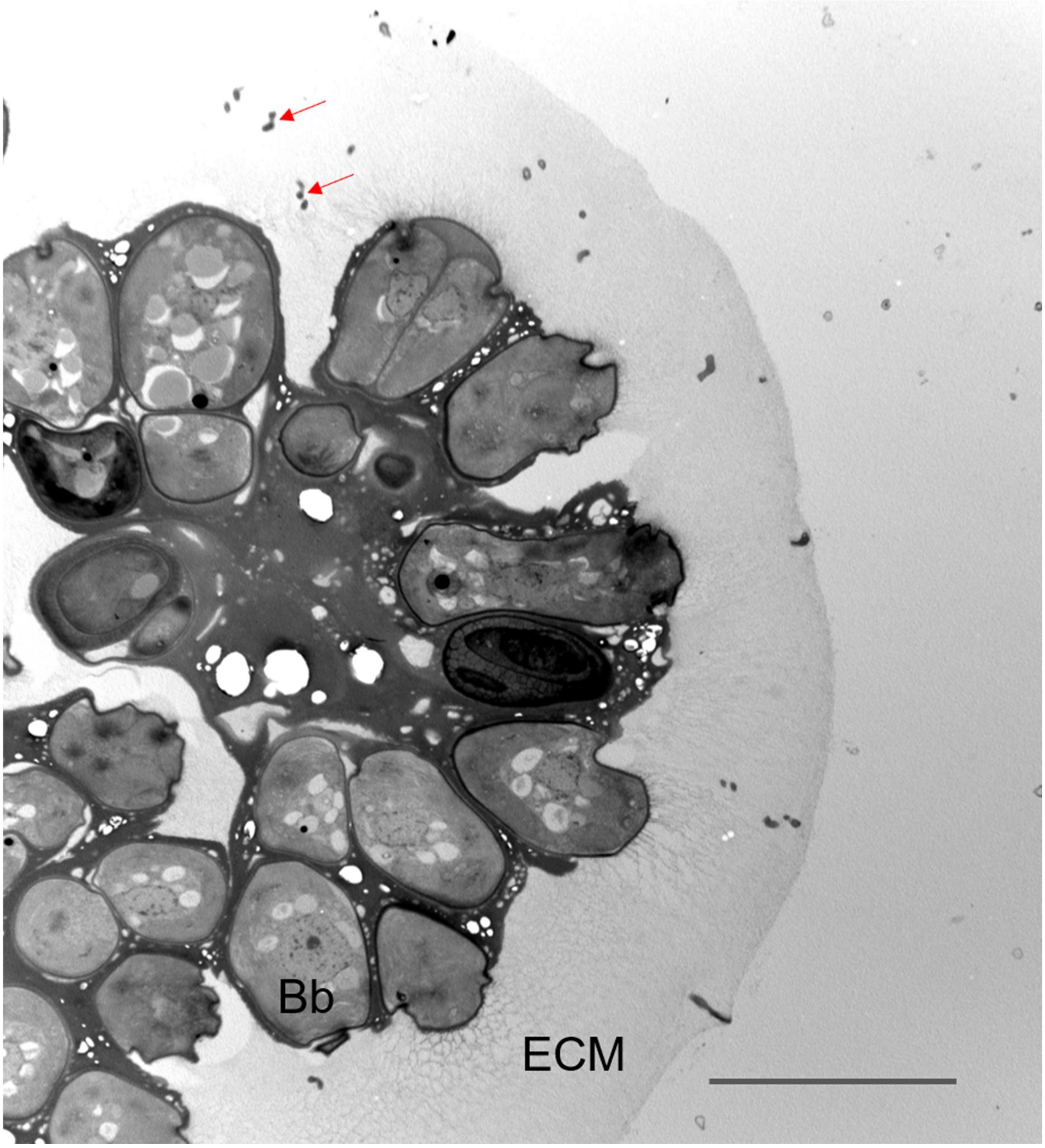

Fig. S2 TEM image of *B. braunii* Ba10. Arrows indicate spiral bacteria. Bb, *B. braunii* cells; ECM, extracellular matrix of *B. braunii*. Scale bar, 10 μm.

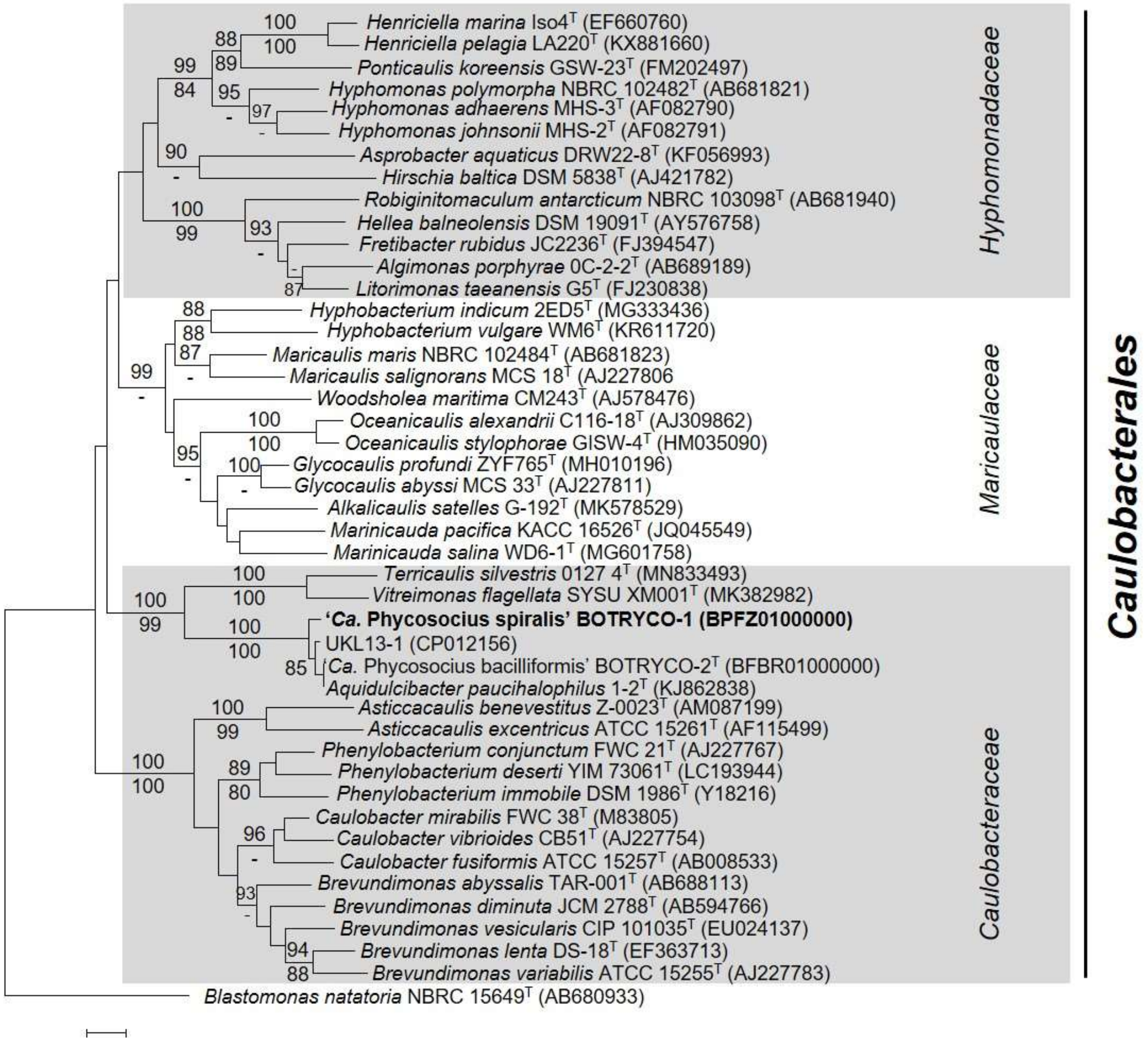

Henriciella marina Iso4T (EF660760)
Henriciella pelagia LA220T (KX881660)
Ponticaulis koreensis GSW-23T (FM202497)
Hyphomonas polymorpha NBRC 102482T (AB681821)
Hyphomonas adhaerens MHS-3T (AF082790)
Hyphomonas johnsonii MHS-2T (AF082791)
Asprobacter aquaticus DRW22-8T (KF056993)
Hirschia baltica DSM 5838T (AJ421782)
Robiginitomaculum antarcticum NBRC 103098T (AB681940)
Hellea balneolensis DSM 19091T (AY576758)
Fretibacter rubidus JC2236T (FJ394547)
Algimonas porphyrae 0C-2-2T (AB689189)
Litorimonas taeanensis G5T (FJ230838)
Hyphobacterium indicum 2ED5T (MG333436)
Hyphobacterium vulgare WM6T (KR611720)
Maricaulis maris NBRC 102484T (AB681823)
Maricaulis salignorans MCS 18T (AJ227806)
Woodsholea maritima CM243T (AJ578476)
Oceanicaulis alexandrii C116-18T (AJ309862)
Oceanicaulis stylophorae GISW-4T (HM035090)
Glycocaulis profundi ZYF765T (MH010196)
Glycocaulis abyssi MCS 33T (AJ227811)
Alkalicaulis satelles G-192T (MK578529)
Marinicauda pacifica KACC 16526T (JQ045549)
Marinicauda salina WD6-1T (MG601758)
Terricaulis silvestris 0127 4T (MN833493)
Vitreimonas flagellata SYSU XM001T (MK382982)
'Ca. Phycosocius spiralis' BOTRYCO-1 (BPFZ01000000)
UKL13-1 (CP012156)
Ca. Phycosocius bacilliformis' BOTRYCO-2T (BFBR01000000)
Aquidulcibacter paucihalophilus 1-2T (KJ862838)
Asticcacaulis benevestitus Z-0023T (AM087199)
Asticcacaulis excentricus ATCC 15261T (AF115499)
Phenylobacterium conjunctum FWC 21T (AJ227767)
Phenylobacterium deserti YIM 73061T (LC193944)
Phenylobacterium immobile DSM 1986T (Y18216)
Caulobacter mirabilis FWC 38T (M83805)
Caulobacter vibrioides CB51T (AJ227754)
Caulobacter fusiformis ATCC 15257T (AB008533)
Brevundimonas abyssalis TAR-001T (AB688113)
Brevundimonas diminuta JCM 2788T (AB594766)
Brevundimonas vesicularis CIP 101035T (EU024137)
Brevundimonas lenta DS-18T (EF363713)
Brevundimonas variabilis ATCC 15255T (AJ227783)
Blastomonas natatoria NBRC 15649T (AB680933)
Hyphomonadaceae
Maricaulaceae
Caulobacteraceae
Caulobacterales

Fig. S3A

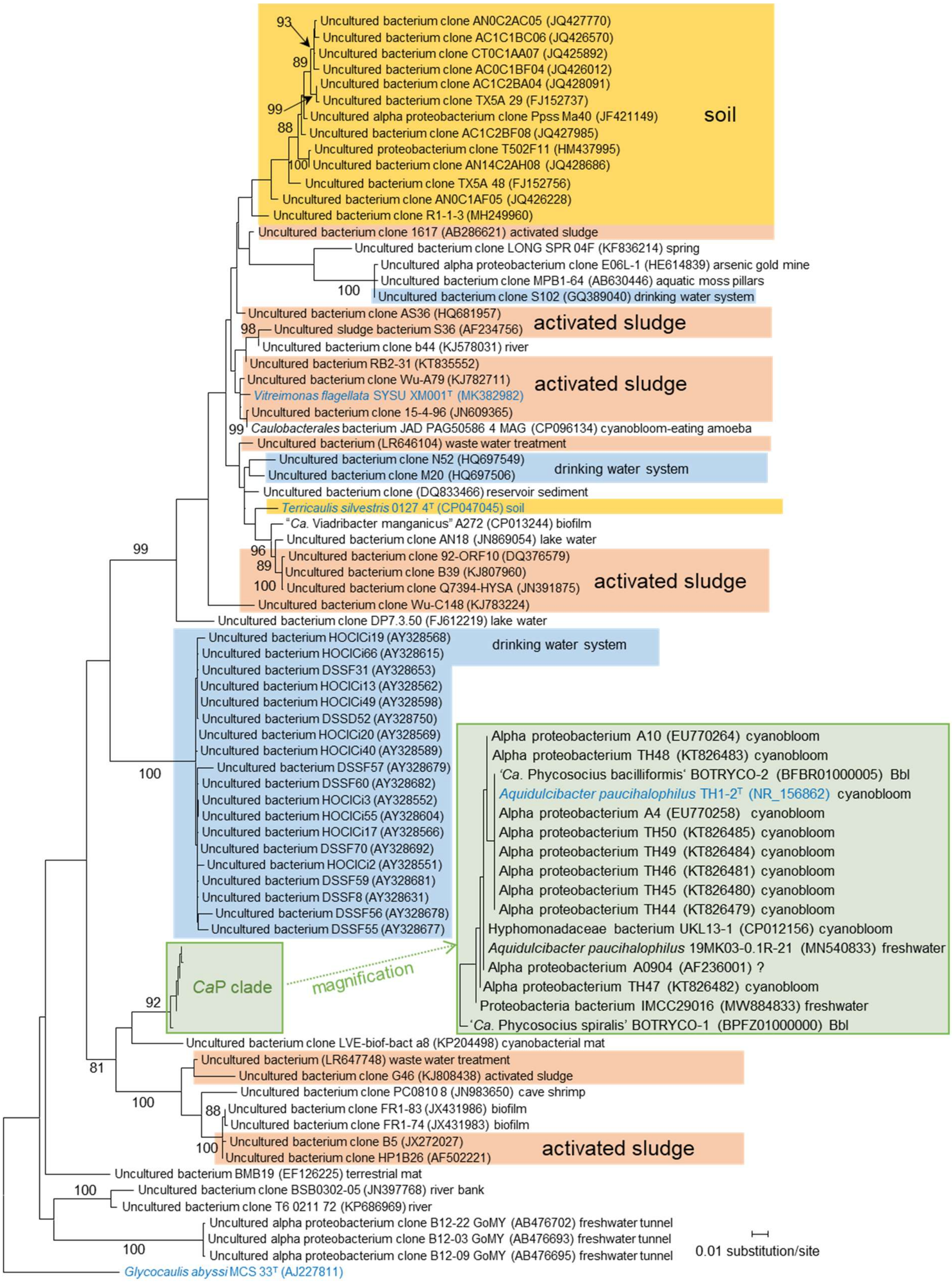
Uncultured bacterium clone AN0C2AC05 (JQ427770)
Uncultured bacterium clone AC1C1BC06 (JQ426570)
Uncultured bacterium clone CT0C1AA07 (JQ425892)
Uncultured bacterium clone AC0C1BF04 (JQ426012)
Uncultured bacterium clone AC1C2BA04 (JQ428091)
Uncultured bacterium clone TX5A 29 (FJ152737)
Uncultured alpha proteobacterium clone Ppss Ma40 (JF421149)
Uncultured bacterium clone AC1C2BF08 (JQ427985)
Uncultured proteobacterium clone T502F11 (HM437995)
Uncultured bacterium clone AN14C2AH08 (JQ428686)
Uncultured bacterium clone TX5A 48 (FJ152756)
Uncultured bacterium clone AN0C1AF05 (JQ426228)
Uncultured bacterium clone R1-1-3 (MH249960)
soil
Uncultured bacterium clone 1617 (AB286621) activated sludge
Uncultured bacterium clone LONG SPR 04F (KF836214) spring
Uncultured alpha proteobacterium clone E06L-1 (HE614839) arsenic gold mine
Uncultured bacterium clone MPB1-64 (AB630446) aquatic moss pillars
Uncultured bacterium clone S102 (GQ389040) drinking water system
Uncultured bacterium clone AS36 (HQ681957)
Uncultured sludge bacterium S36 (AF234756)
activated sludge
Uncultured bacterium clone b44 (KJ578031) river
Uncultured bacterium RB2-31 (KT835552)
Uncultured bacterium clone Wu-A79 (KJ782711)
Vitreimonas flagellata SYSU XM001^{T} (MK382982)
Uncultured bacterium clone 15-4-96 (JN609365)
activated sludge
Caulobacterales bacterium JAD PAG50586 4 MAG (CP096134) cyanobloom-eating amoeba
Uncultured bacterium (LR646104) waste water treatment
Uncultured bacterium clone N52 (HQ697549)
Uncultured bacterium clone M20 (HQ697506)
drinking water system
Uncultured bacterium clone (DQ833466) reservoir sediment
Terricaulis silvestris 0127 4^{T} (CP047045) soil
"Ca. Viadribacter manganicus" A272 (CP013244) biofilm
Uncultured bacterium clone AN18 (JN869054) lake water
Uncultured bacterium clone 92-ORF10 (DQ376579)
Uncultured bacterium clone B39 (KJ807960)
Uncultured bacterium clone Q7394-HYSA (JN391875)
activated sludge
Uncultured bacterium clone Wu-C148 (KJ783224)
Uncultured bacterium clone DP7.3.50 (FJ612219) lake water
Uncultured bacterium HOCICi19 (AY328568)
drinking water system
Uncultured bacterium HOCICi66 (AY328615)
Uncultured bacterium DSSF31 (AY328653)
Uncultured bacterium HOCICi13 (AY328562)
Uncultured bacterium HOCICi49 (AY328598)
Uncultured bacterium DSSD52 (AY328750)
Uncultured bacterium HOCICi20 (AY328569)
Uncultured bacterium HOCICi40 (AY328589)
Uncultured bacterium DSSF57 (AY328679)
Uncultured bacterium DSSF60 (AY328682)
Uncultured bacterium HOCICi3 (AY328552)
Uncultured bacterium HOCICi55 (AY328604)
Uncultured bacterium HOCICi17 (AY328566)
Uncultured bacterium DSSF70 (AY328692)
Uncultured bacterium HOCICi2 (AY328551)
Uncultured bacterium DSSF59 (AY328681)
Uncultured bacterium DSSF8 (AY328631)
Uncultured bacterium DSSF56 (AY328678)
Uncultured bacterium DSSF55 (AY328677)
Alpha proteobacterium A10 (EU770264) cyanobloom
Alpha proteobacterium TH48 (KT826483) cyanobloom
'Ca. Phycosocius bacilliformis' BOTRYCO-2 (BFBR01000005) Bbl
Aquidulcibacter paucihalophilus TH1-2^{T} (NR_156862) cyanobloom
Alpha proteobacterium A4 (EU770258) cyanobloom
Alpha proteobacterium TH50 (KT826485) cyanobloom
Alpha proteobacterium TH49 (KT826484) cyanobloom
Alpha proteobacterium TH46 (KT826481) cyanobloom
Alpha proteobacterium TH45 (KT826480) cyanobloom
Alpha proteobacterium TH44 (KT826479) cyanobloom
Hyphomonadaceae bacterium UKL13-1 (CP012156) cyanobloom
Aquidulcibacter paucihalophilus 19MK03-0.1R-21 (MN540833) freshwater
Alpha proteobacterium A0904 (AF236001) ?
Alpha proteobacterium TH47 (KT826482) cyanobloom
Proteobacteria bacterium IMCC29016 (MW884833) freshwater
'Ca. Phycosocius spiralis' BOTRYCO-1 (BPFZ01000000) Bbl
CaP clade
magnification
Uncultured bacterium clone LVE-biof-bact a8 (KP204498) cyanobacterial mat
Uncultured bacterium (LR647748) waste water treatment
Uncultured bacterium clone G46 (KJ808438) activated sludge
Uncultured bacterium clone PC0810 8 (JN983650) cave shrimp
Uncultured bacterium clone FR1-83 (JX431986) biofilm
Uncultured bacterium clone FR1-74 (JX431983) biofilm
Uncultured bacterium clone B5 (JX272027)
Uncultured bacterium clone HP1B26 (AF502221)
activated sludge
Uncultured bacterium BMB19 (EF126225) terrestrial mat
Uncultured bacterium clone BSB0302-05 (JN397768) river bank
Uncultured bacterium clone T6 0211 72 (KP686969) river
Uncultured alpha proteobacterium clone B12-22 GoMY (AB476702) freshwater tunnel
Uncultured alpha proteobacterium clone B12-03 GoMY (AB476693) freshwater tunnel
Uncultured alpha proteobacterium clone B12-09 GoMY (AB476695) freshwater tunnel
Glycocaulis abyssi MCS 33^{T} (AJ227811)
0.01 substitution/site

Fig. S3B

Fig. S3 16S rDNA phylogenetic analysis. A. Phylogenetic tree containing only type species (plus UKL-13). An alignment (1 119 bp) without gaps was generated using Clustal W. 16S rDNA phylogenetic analyses were performed using MEGA ver. 7 (Kumar *et al.*, 2017) and RaxML-NG ver. 1.0.1 for distance and ML phylogenetic reconstructions, respectively. Maximum composite likelihood distance calculation based on gamma distributed site-rate variation model was used for neighbor-joining (NJ) tree reconstruction. ML phylogenetic reconstruction was performed based on DNA substitution model 'GTR+I+G4' inferred using Modeltest-NG. Bootstrap values based on 1 000 replicates are indicated above (NJ) and below (ML) each branch. **B.** Phylogenetic tree including uncharacterized strains and uncultured clones. An alignment (1 254 bps) without gaps was generated using Clustal W. The tree was constructed using RaxML-NG on the basis of the DNA substitution model 'GTR+I+G4' inferred using Modeltest-NG. ML bootstrap values (>80%) on the basis of 1 000 replicates are indicated at each branch. Taxa validly published under the International Code of Nomenclature of Prokaryotes (ICNP) are indicated in blue. Isolation sources are indicated after parentheses or inside the colored boxes. The green box indicates the *Ca*P clade, in which all members were recovered or isolated from phytoplankton, except for three sequences recovered from undescribed freshwater sources. Bbl, *Botryococcus* bloom.

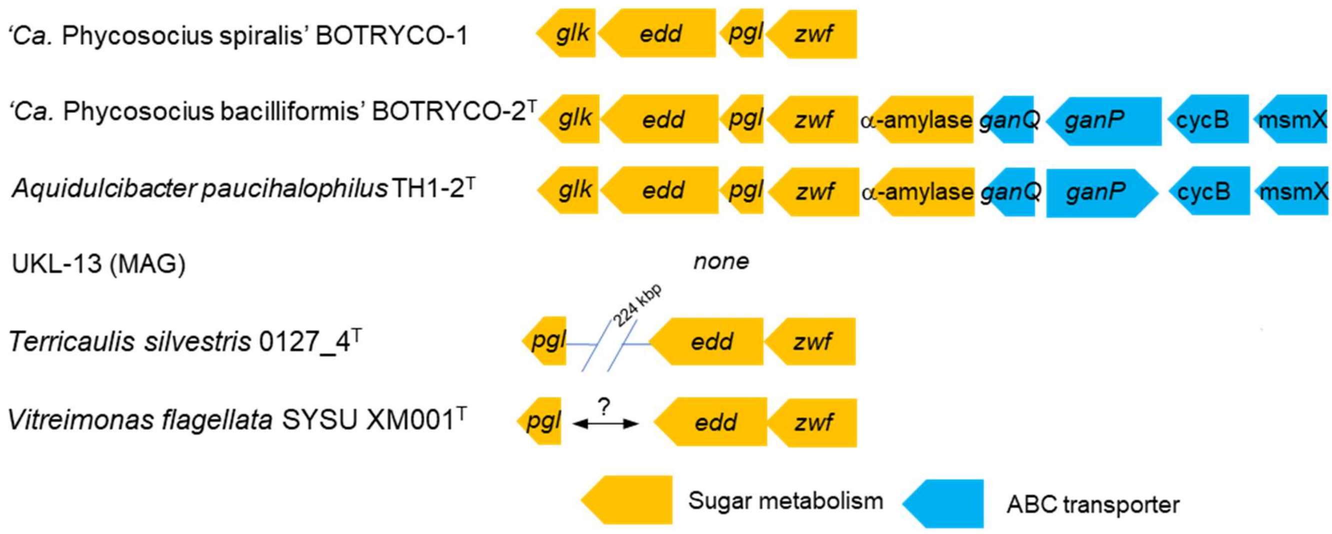

Fig. S4 Organization of genes for galactose metabolism. Genes in the orange box are not directly involved in galactose metabolism except for α-amylase, which is possibly involved in galactose oligomer breakdown.

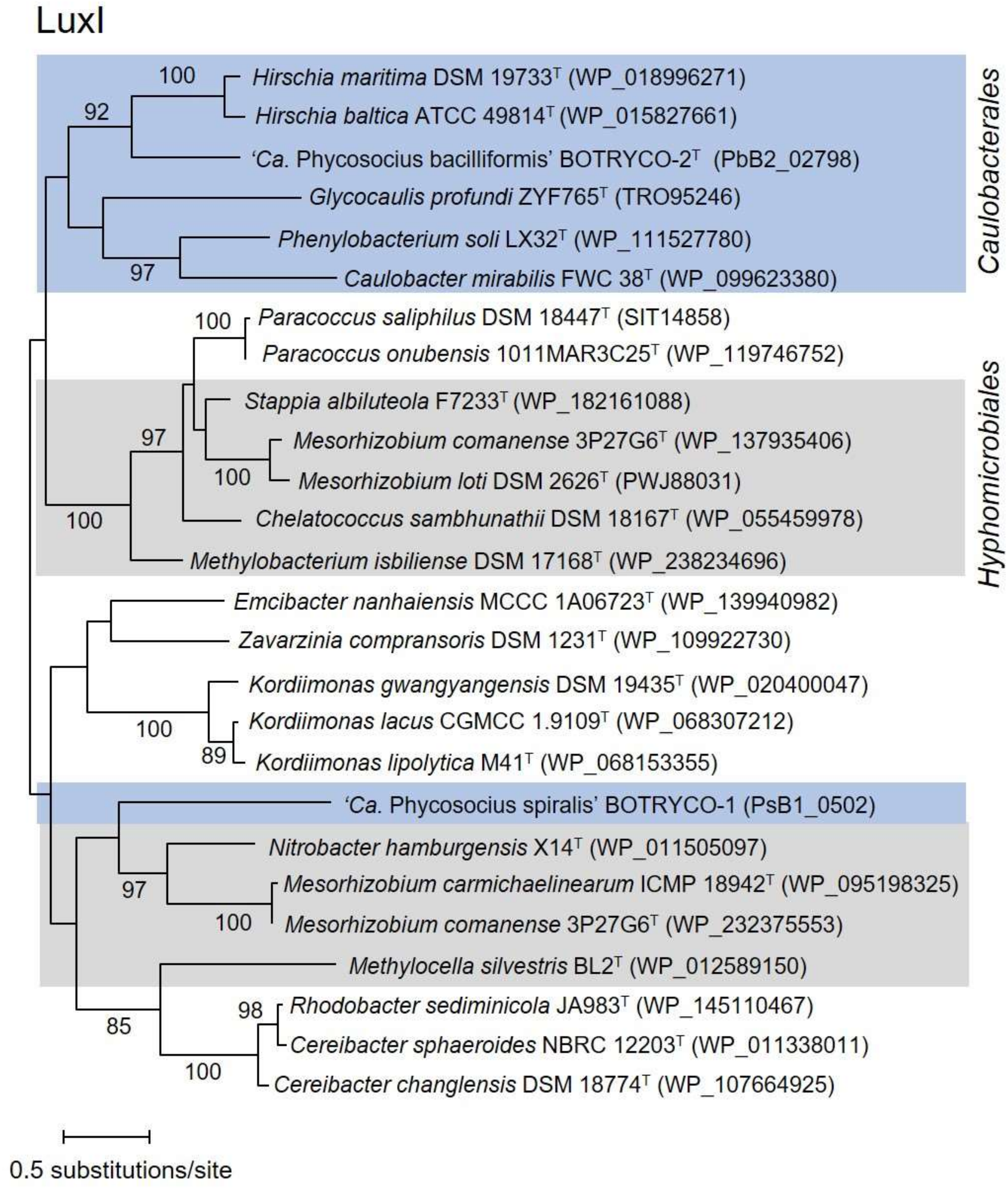
LuxI
100
92
Hirschia maritima DSM 19733T (WP_018996271)
Hirschia baltica ATCC 49814T (WP_015827661)
'Ca. Phycosocius bacilliformis' BOTRYCO-2T (PbB2_02798)
Glycocaulis profundi ZYF765T (TRO95246)
Phenylobacterium soli LX32T (WP_111527780)
97
Caulobacter mirabilis FWC 38T (WP_099623380)
Caulobacterales
100
Paracoccus saliphilus DSM 18447T (SIT14858)
Paracoccus onubensis 1011MAR3C25T (WP_119746752)
Stappia albiluteola F7233T (WP_182161088)
97
Mesorhizobium comanense 3P27G6T (WP_137935406)
100
Mesorhizobium loti DSM 2626T (PWJ88031)
100
Chelatococcus sambhunathii DSM 18167T (WP_055459978)
Methylobacterium isbiliense DSM 17168T (WP_238234696)
Hyphomicrobiales
Emcibacter nanhaiensis MCCC 1A06723T (WP_139940982)
Zavarzinia compransoris DSM 1231T (WP_109922730)
Kordiimonas gwangyangensis DSM 19435T (WP_020400047)
100
Kordiimonas lacus CGMCC 1.9109T (WP_068307212)
89
Kordiimonas lipolytica M41T (WP_068153355)
'Ca. Phycosocius spiralis' BOTRYCO-1 (PsB1_0502)
Nitrobacter hamburgensis X14T (WP_011505097)
97
Mesorhizobium carmichaelinearum ICMP 18942T (WP_095198325)
100
Mesorhizobium comanense 3P27G6T (WP_232375553)
Methylocella silvestris BL2T (WP_012589150)
98
Rhodobacter sediminicola JA983T (WP_145110467)
85
Cereibacter sphaeroides NBRC 12203T (WP_011338011)
100
Cereibacter changlensis DSM 18774T (WP_107664925)
0.5 substitutions/site

Fig. S5A

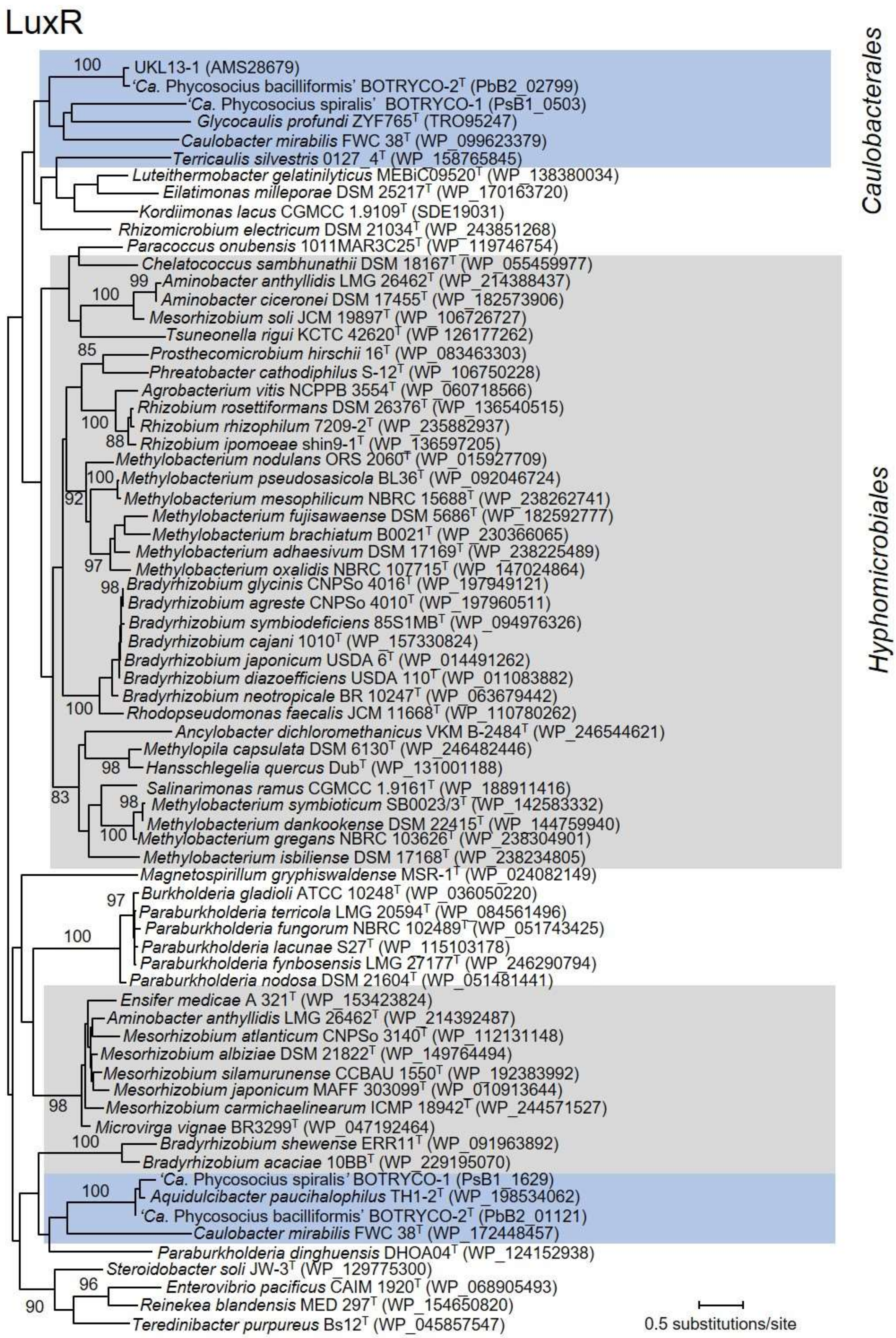
LuxR
Caulobacterales
Hyphomicrobiales
UKL13-1 (AMS28679)
'Ca. Phycosocius bacilliformis' BOTRYCO-2T (PbB2_02799)
'Ca. Phycosocius spiralis' BOTRYCO-1 (PsB1_0503)
Glycocaulis profundi ZYF765T (TRO95247)
Caulobacter mirabilis FWC 38T (WP_099623379)
Terricaulis silvestris 0127_4T (WP_158765845)
Luteithermobacter gelatinilyticus MEBiC09520T (WP_138380034)
Eilatimonas milleporae DSM 25217T (WP_170163720)
Kordiimonas lacus CGMCC 1.9109T (SDE19031)
Rhizomicrobium electricum DSM 21034T (WP_243851268)
Paracoccus onubensis 1011MAR3C25T (WP_119746754)
Chelatococcus sambhunathii DSM 18167T (WP_055459977)
Aminobacter anthyllidis LMG 26462T (WP_214388437)
Aminobacter ciceronei DSM 17455T (WP_182573906)
Mesorhizobium soli JCM 19897T (WP_106726727)
Tsuneonella rigui KCTC 42620T (WP 126177262)
Prosthecomicrobium hirschii 16T (WP_083463303)
Phreatobacter cathodiphilus S-12T (WP_106750228)
Agrobacterium vitis NCPPB 3554T (WP_060718566)
Rhizobium rosettiformans DSM 26376T (WP_136540515)
Rhizobium rhizophilum 7209-2T (WP_235882937)
Rhizobium ipomoeae shin9-1T (WP_136597205)
Methylobacterium nodulans ORS 2060T (WP_015927709)
Methylobacterium pseudosasicola BL36T (WP_092046724)
Methylobacterium mesophilicum NBRC 15688T (WP_238262741)
Methylobacterium fujisawaense DSM 5686T (WP_182592777)
Methylobacterium brachiatum B0021T (WP_230366065)
Methylobacterium adhaesivum DSM 17169T (WP_238225489)
Methylobacterium oxalidis NBRC 107715T (WP_147024864)
Bradyrhizobium glycinis CNPSo 4016T (WP_197949121)
Bradyrhizobium agreste CNPSo 4010T (WP_197960511)
Bradyrhizobium symbiodeficiens 85S1MBT (WP_094976326)
Bradyrhizobium cajani 1010T (WP_157330824)
Bradyrhizobium japonicum USDA 6T (WP_014491262)
Bradyrhizobium diazoefficiens USDA 110T (WP_011083882)
Bradyrhizobium neotropicale BR 10247T (WP_063679442)
Rhodopseudomonas faecalis JCM 11668T (WP_110780262)
Ancylobacter dichloromethanicus VKM B-2484T (WP_246544621)
Methylopila capsulata DSM 6130T (WP_246482446)
Hansschlegelia quercus DubT (WP_131001188)
Salinarimonas ramus CGMCC 1.9161T (WP_188911416)
Methylobacterium symbioticum SB0023/3T (WP_142583332)
Methylobacterium dankookense DSM 22415T (WP_144759940)
Methylobacterium gregans NBRC 103626T (WP_238304901)
Methylobacterium isbiliense DSM 17168T (WP_238234805)
Magnetospirillum gryphiswaldense MSR-1T (WP_024082149)
Burkholderia gladioli ATCC 10248T (WP_036050220)
Paraburkholderia terricola LMG 20594T (WP_084561496)
Paraburkholderia fungorum NBRC 102489T (WP_051743425)
Paraburkholderia lacunae S27T (WP_115103178)
Paraburkholderia fynbosensis LMG 27177T (WP_246290794)
Paraburkholderia nodosa DSM 21604T (WP_051481441)
Ensifer medicae A 321T (WP_153423824)
Aminobacter anthyllidis LMG 26462T (WP_214392487)
Mesorhizobium atlanticum CNPSo 3140T (WP_112131148)
Mesorhizobium albiziae DSM 21822T (WP_149764494)
Mesorhizobium silamurunense CCBAU 1550T (WP_192383992)
Mesorhizobium japonicum MAFF 303099T (WP_010913644)
Mesorhizobium carmichaelinearum ICMP 18942T (WP_244571527)
Microvirga vignae BR3299T (WP_047192464)
Bradyrhizobium shewense ERR11T (WP_091963892)
Bradyrhizobium acaciae 10BBT (WP_229195070)
'Ca. Phycosocius spiralis' BOTRYCO-1 (PsB1_1629)
Aquidulcibacter paucihalophilus TH1-2T (WP_198534062)
'Ca. Phycosocius bacilliformis' BOTRYCO-2T (PbB2_01121)
Caulobacter mirabilis FWC 38T (WP_172448457)
Paraburkholderia dinghuensis DHOA04T (WP_124152938)
Steroidobacter soli JW-3T (WP_129775300)
Enterovibrio pacificus CAIM 1920T (WP_068905493)
Reinekea blandensis MED 297T (WP_154650820)
Teredinibacter purpureus Bs12T (WP_045857547)
100
99
100
85
100
88
100
92
97
98
100
98
83
98
100
97
100
98
100
100
96
90
0.5 substitutions/site

Fig. S5B

Fig. S5 Molecular phylogeny of QS proteins. Midpoint rooted ML phylogenetic trees of LuxI (A) and LuxR (B). QS protein sequences related to *Ca*P bacteria were retrieved from GenBank. Phylogenetic reconstructions were according to the methods as described in the main text. LuxI and LuxR trees were generated based on 'LG+I+G4+F' and 'LG+G4+F', respectively, using gap-free alignments (162 and 172 amino acids, respectively). ML bootstrap values (>80%) based on 1 000 replicates are indicated at each branch. Note that LuxR of '*Ca*. P. spiralis' (PsB1_0503) clusters with those of other *Ca*P bacteria, whereas its putative ligand LuxI (PsB1_0502) is distantly related to LuxI of '*Ca*. P. bacilliformis' (PbB2_02798), suggesting HGT.

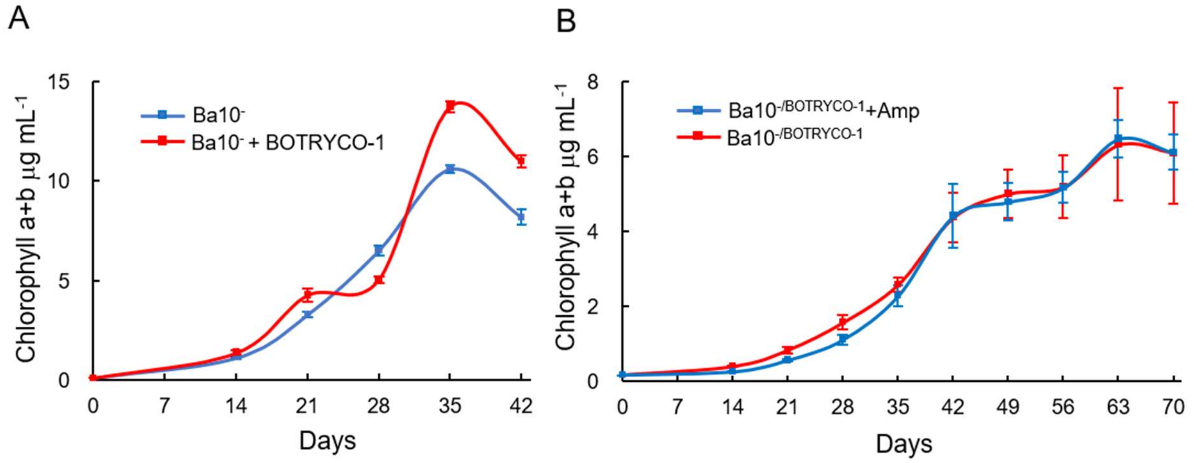

Fig. S6 Growth of *B. braunii* with or without BOTRYCO-1. A Growth of axenic *B. braunii* Ba10 (denoted Ba10^{-}) with or without inoculation of BOTRYCO-1. **B** Growth of *B. braunii* Ba10^{-} maintained with BOTRYCO-1 in more than three subcultures (denoted Ba10$^{-/BOTRYCO-1}$) with or without ampicillin treatment (at a final concentration of 50 μg/mL). After 14 days, BOTRYCO-1 was not observed in the ampicillin-treated culture. Bars indicate the standard error of three biological replicates. Protocols for culture, bacteria inoculation, and chlorophyll extraction were according to [15].

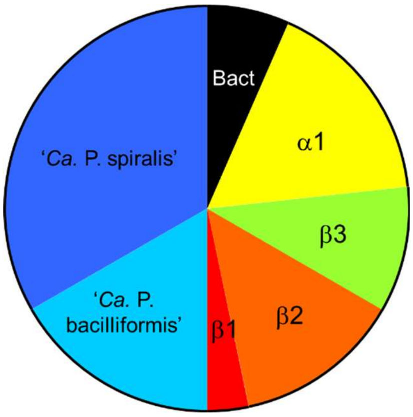

Abbr.	Top BLAST hit (type strain)	Similarity (%)
Bact	*Chryseotalea sanaruensis* Ys^{T}	88.1
α1	*Azospirillum rugosum* IMMIB AFH-6^{T}	91.4
β3	*Pelocola oligotropha* An3-4^{T}	93.0
β2	*Rhodoferax lacus* IMCC26218^{T}	98.9
β1	*Rhodoferax lacus* IMCC26218^{T}	99.0

Fig. S7 Microbiome of *B. braunii* Ba10. Bacterial species composition of a non-axenic culture of *B. braunii* Ba10 in liquid AF-6 medium was inferred using 16S rDNA clone library analyses. 16S rDNA were PCR amplified using the universal primer pair of 27F/1492R (Lane *et al.*, 1991) and clone libraries were constructed using a Zero Blunt TOPO PCR Cloning Kit (Invitrogen, Carlsbad, CA). More than 30 clones were Sanger sequenced. The 30 clones remaining after the removal of errors and possible artificial sequences originating from PCR chimeras are shown in the pie chart. BLAST results were as of 01/04/2023. Attempts to culture bacteria using TSA and NA media were unsuccessful except for ‘*Ca*. P. bacilliformis’ [15]. Nucleotide sequence data are available under the DDBJ accession numbers LC745732–LC745736.

Supplementary method

Pigment analyses

Collected BOTRYCO-1 cells were mixed with 500 μL of methanol in a 1.5 mL microtube and vortexed for 10 s. After centrifugation at 20 000 g, absorbance (within the spectral range of 350–900 nm) was measured using a UV spectrophotometer UV-1800 (Shimadzu, Kyoto, Japan).

Movie S1 Swimming motility of '*Ca.* P. spiralis' (.wmv)

Movie S2 Colonization of '*Ca.* P. spiralis' inside the ECM of *B. braunii* (.wmv)

References

Kim M, Shin B, Lee J, Park HY, Park W. Culture-independent and culture-dependent analyses of the bacterial community in the phycosphere of cyanobloom-forming *Microcystis aeruginosa*. Sci Rep. 2019; 9:20416

Kumar S, Stecher G, Tamura K. MEGA7: Molecular Evolutionary Genetics Analysis Version 7.0 for Bigger Datasets. Mol Biol Evol. 2016; 33:1870–1874.

Lane DJ. 16S/23S rRNA sequencing. In: Stackebrandt E & Goodfellow M (eds.) Nucleic acid techniques in bacterial systematics. John Wiley and Sons, 1991. pp 115–175.

Li Q, Lin F, Yang C, Wang J, Lin Y, Shen M, et al. A large-scale comparative metagenomic study reveals the functional interactions in six bloom-forming *Microcystis*-epibiont communities. Front Microbiol. 2018; 9:746

Parulekar NN, Kolekar P, Jenkins A, Kleiven S, Utkilen H, Johansen A, et al. Characterization of bacterial community associated with phytoplankton bloom in a eutrophic lake in South Norway using 16S rRNA gene amplicon sequence analysis. PLoS ONE 2017; 12:e0173408

Tromas N, Fortin N, Bedrani L, Terrat Y, Cardoso P, Bird D, et al. Characterising and predicting cyanobacterial blooms in an 8-year amplicon sequencing time course. ISME J. 2017; 11:1746-1763
